# Glyoxal-derived advanced glycation end products (GO-AGEs) with UVB critically induce skin inflammaging: in vitro and in silico approaches

**DOI:** 10.1038/s41598-024-52037-z

**Published:** 2024-01-22

**Authors:** Razia Sultana, Amna Parveen, Min-Cheol Kang, Seong-Min Hong, Sun Yeou Kim

**Affiliations:** 1 Department of Pharmacy, Jagannath University, Dhaka, 1100 Bangladesh; 2 Department of Life Science, University of Seoul, Seoul, 02504 Korea; 3 College of Pharmacy, Gachon University, #191, Hambakmoero, Yeonsu-gu, Incheon, 21936 Korea; 4 MetaCen Therapeutics Company, # Changnyong-daero 256 beon-gil, Yeongtong-gu, Suwon-si, Gyeonggi-do 16229 Republic of Korea; 5 Gachon Institute of Pharmaceutical Science, Gachon University, #191, Hambakmoe-ro, Yeonsu-gu, Incheon, 21936 Republic of Korea

**Keywords:** Cell biology, Chemical biology, Computational biology and bioinformatics, Molecular biology, Molecular medicine

## Abstract

Advanced glycation end products (AGEs) have potential implications on several diseases including skin inflammation and aging. AGEs formation can be triggered by several factors such as UVB, glyoxal and methylglyoxal etc. However, little attention has been paid to glyoxal-derived AGEs (GO-AGEs) and UVB-induced skin inflammaging, with none have investigated together. This study aimed to investigate the possible role of GO-AGEs and UVB in skin inflammaging focusing on revealing its molecular mechanisms. The effects of GO-AGEs in the presence or absence of UVB were studied by using enzyme linked immunosorbent assay, western blotting, qPCR, flow cytometry and in silico approaches. In HaCaT cells, GO-AGEs in the presence of UVB irradiation (125 mJ/cm^2^) dramatically enhanced the release of different pro-inflammatory cytokines (IL-1β, IL-6, and TNF-α) with further activation of RAGE signaling pathways (NF-κB, COX 2, and IL- 1β) and increased oxidative stress also noticed in NHEK cells. In NHDF cells, extracellular matrix disruption noted via increasing matrix metalloproteinase release and decreasing collagen type 1 and SIRT1 expression. Besides that, the docking scores obtained from the molecular docking study support the above-mentioned results. This study strongly suggests the pivotal role of GO-AGEs in skin inflammaging and illuminates novel molecular pathways for searching most effective and updated anti-aging therapy.

## Introduction

The skin, composed of epidermis and dermis, is the largest external barrier and protects against physical, chemical, and biological insults^1^. Skin aging is a complex biological process involving histological and morphological changes caused by intrinsic (e.g., genetic, and endogenous) and extrinsic (e.g., UV rays, and contaminants) factors^2^. It is accompanied by dry skin, loss of elasticity, wrinkles, discoloration, delayed wound healing, and the development of several benign and malignant diseases^3^. Inflammation is one of the major and primitive causes of intrinsic and extrinsic aging, as the innate and adaptive immune systems are activated by external and internal stimuli, respectively^4^. The aging process is accompanied by consistent low-grade, asymptomatic, and chronic inflammation, referred to as inflammaging^5^. Inflammation has been linked to several aging-related disorders with significant morbidity and mortality, including skin aging, atherosclerosis, diabetes, and Alzheimer's disease^6^.

Recent studies have focused on AGEs (advanced glycation end-products) as pathophysiological factor linked to oxidative stress, inflammation, aging, metabolic disorders, and cardiovascular diseases^7,8^. AGEs have been implicated in various dermatological conditions, including skin inflammation, psoriasis, atopic dermatitis, melanogenesis, and wound healing^9^. The accumulation of AGEs is characterized by glycated keratin of yellowish color and dry skin, which are both early indicators of AGEs-related pathological conditions, including skin cancers^10^. Several pathological conditions (diabetes, and hyperglycemia), smoking, and ROS aggravating factors, lead to the formation of different reactive di-carbonyl compounds, including methylglyoxal (MGO), glyoxal (GO), and 3-deoxyglucosone (3DG), which facilitate AGEs formation. Furthermore, these compounds are involved in anabolic glycolysis, lipid peroxidation, and DNA modification via the Maillard reaction (i.e., non-enzymatic glycation/oxidation of proteins, lipids, and nucleic acids, that form covalent bonds with reducing sugars) to produce AGEs. Certain identical foods act as an external source of AGEs, including dry-heated foods, baked goods, edible oils, etc.^11,12^. Based on molecular mechanisms, AGES interacts with the transmembrane receptors for AGEs (RAGE)^13^, which further activates the inflammatory signaling pathway and releases pro-inflammatory cytokines (IL-1 β, IL-6), followed by the production of matrix metalloproteinase (MMP) and the degradation of collagen and other components of the dermal extracellular matrix (ECM), thereby ultimately facilitating the aging process^14^.

Among various exogenous factors, ultraviolet (UV) irradiation is considered as the most harmful, energetic, and carcinogenic, causing skin disorders such as sunburn, photoaging, pigmentation, and photocarcinogenesis^15^. UVRs can induce DNA damage, oxidative stress, and inflammation by inducing several inflammatory cytokines such as Interleukin-1 alpha (IL-1α), Interleukin-1 beta (IL-1β), Interleukin-6 (IL-6), and Tumor Necrosis Factor-alpha (TNF-α) which further causes aggravation of caspase 1 and Mitogen-Activated Protein Kinases (MAPK) signaling pathways^16^. Reportedly, UVB irradiation also can induce AGEs formation^17^ and is linked to photoaging, wrinkling, skin pigmentation, and increase susceptibility to skin cancer^18^. Several studies have reported that intracellular AGEs formation by UVB irradiation induces the formation of ROS and triggers inflammatory signaling responses that damage several proteins upon binding with RAGE^19,20^. Different reactive intermediates of glycolysis such as GO-derived AGEs have been reported to impair wound healing, disrupting the skin’s integrity^21,22^. In addition, it is involved in the degradation of dermal collagen and the elastin in ECM, which ultimately contributes to stiffness, loss of elasticity, and wrinkle formation, which facilitate skin aging^23^. Interestingly, GO and/or MGO, the reactive dicarbonyl endogenous metabolites, are reported to be present in exogenous sources such as food, beverages, and cigarette smoke^11,12^. However few studies have been performed on GO-derived AGEs, with most related to the structural identification and quantification of GO-AGEs, mainly using glyoxal- lysine dimer (GOLD)^24^. As a novel ligand and a representative group of GO-derived AGEs, GOLD has already been demonstrated to interact with several cell signaling molecules, revealing molecular mechanisms, as reported via in silico^25^ and in-vitro cell studies^26^.

To date, no study till now have been conducted on GO-derived AGEs in skin aging and rarely in UVB-induced inflammaging. Concerning the above information, this study was designed to investigate the effect of GO-AGEs in skin inflammation under normal and UVB- irradiated conditions in vitro.

## Results

### Cytotoxic effects of GO-AGEs in human epidermal keratinocytes and dermal fibroblast cells

**Figure 1:**
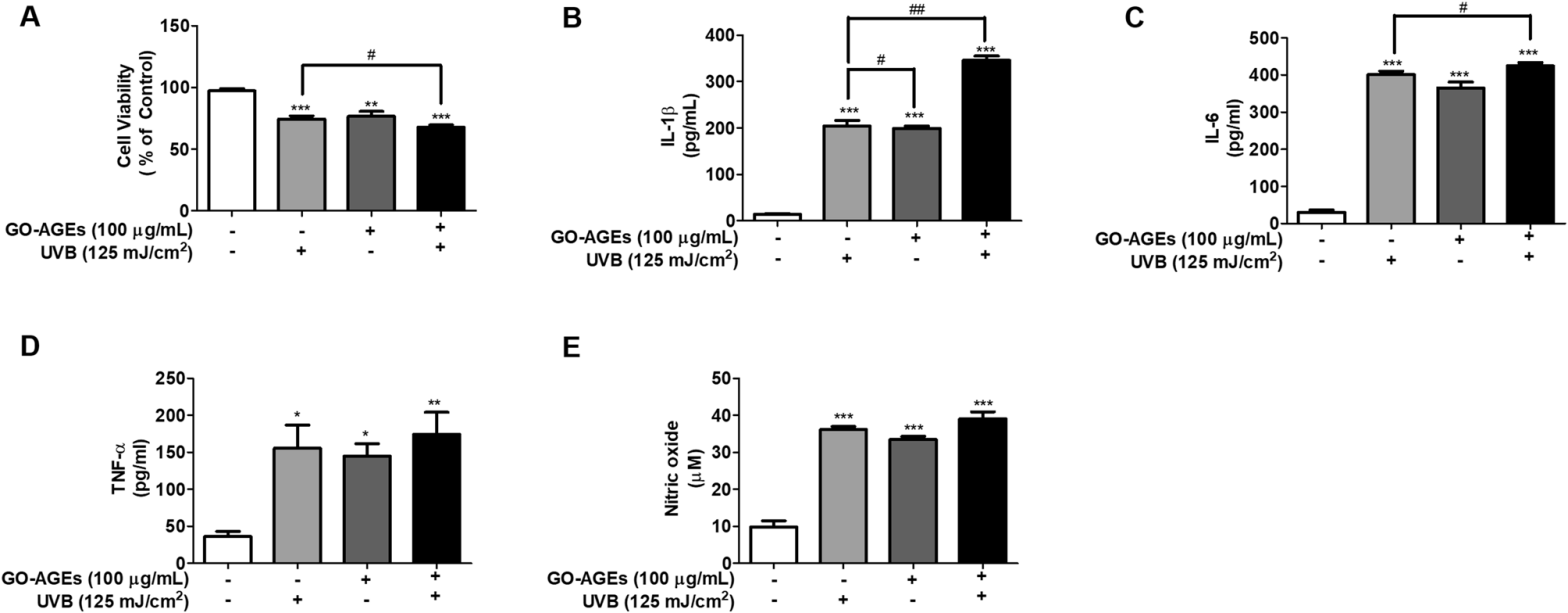
GO-AGEs induce the elevated secretion of different pro-inflammatory cytokines in HaCaT cells. (**A**) MTT assay showing the effects of GO-AGEs (100 μg/mL) in the presence or absence of UVB irradiation (125 mJ/cm^2^), along with the control group in HaCaT cells. (**B**–**D**) ELISA results demonstrating the elevated proinflammatory cytokine levels of IL-1β (**B**), IL-6 (**C**), and TNF-α (**D**) in the conditioned medium of HaCaT cells induced by GO-AGEs in the presence or absence of UVB irradiation. (**E**) The nitric oxide (NO) level in HaCaT cells was detected via NO assay in the conditioned medium of HaCaT cells as induced by GO-AGEs in the presence or absence of UVB irradiation. Each value represents the mean ± SEM of triplicate experiments. *p < 0.05, **p < 0.01, and ***p < 0.001 vs. the control group and ^#^p < 0.05 and ^##^p < 0.01 vs. the UVB treatment group induced by UVB irradiation (125 mJ/cm^2^).

Based on the cytotoxic effects evaluated by MTT assay, it is evident that GO-AGEs, UVB, and GO-AGEs + UVB treatment groups had notable inhibition on the cell viability than that in the control group. Interestingly, the cell viability was noticed to follow a similar decreasing pattern in all three cell lines (HaCaT, NHEK, and NHDF cells) as demonstrated in Figs. 1A, 3A, and 4A. However, the GO-AGEs + UVB treatment group significantly diminished cell viability in all three cell lines in comparison to the other groups.

### GO-AGEs induce pro-inflammatory cytokines secretion and NO release in HaCaT cells

**Figure 2:**
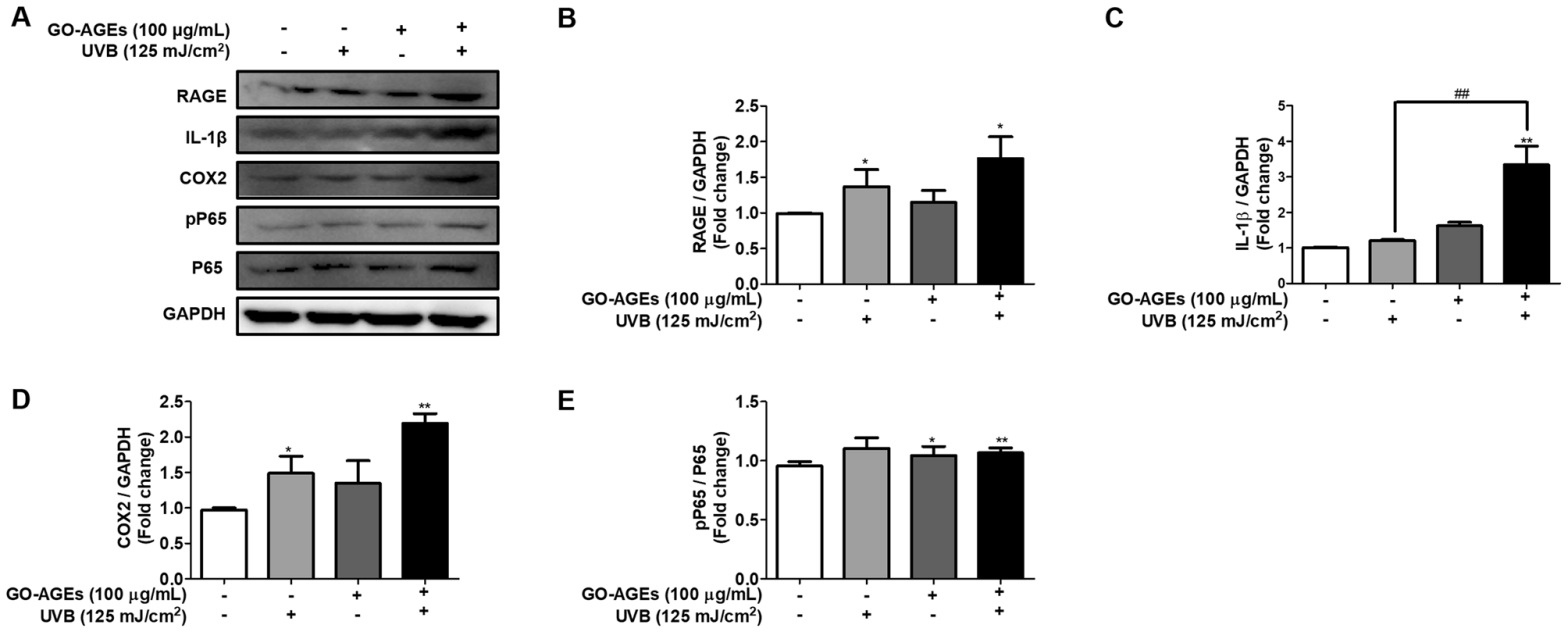
GO-AGEs upregulate the expression of RAGE and its downstream-signaling pathway related proteins. (**A**) Western blot analysis showing the upregulated expression of RAGE and its downstream signaling pathway related inflammatory proteins, particularly IL-1β, COX2, pP65, and P65. Upon treatment with GO-AGEs (100 μg/mL) in the presence or absence of UVB irradiation (125 mJ/cm^2^), along with the control group in HaCaT cells. (**B**–**E**) Quantification of the expression levels of RAGE (**B**), IL-1β (**C**), COX2 (**D**), pP65/P65 (**E**) proteins. Each value represents the mean ± SEM of triplicate experiments. *p < 0.05 and **p < 0.01 vs. the control group and ^##^p < 0.01 vs. the UVB treatment group induced by UVB irradiation (125 mJ/cm^2^).

The treatment groups of GO-AGEs, UVB, and GO-AGEs + UVB have been observed to significantly induce the release of different pro-inflammatory cytokines (IL-1β, IL-6, and TNF-α), as shown in Fig. 1B–D evaluated by using ELISA. Based on the obtained ELISA results, the concentrations of IL-1β (Fig. 1B) and TNF-α (Fig. 1D) was increased significantly in the cell supernatant by GO-AGEs + UVB treatment group, as compared to the GO-AGEs and UVB group individually. In addition, the up-regulation of IL-1β at the protein level was also observed in that particular GO-AGEs + UVB treatment group (Fig. 2A,C). Mention- worthy, GO-AGEs combined with UVB group enhanced IL-1β activation by approximately three fold than the control group. Furthermore, there was an substantial elevation in the NO level when treated with GO-AGEs group, which was further augmented in the presence of UVB irradiation in comparison to that in other groups in HaCaT cells as shown in Fig. 1E.

### GO-AGEs increase RAGE expression and its downstream-inflammatory signaling pathway in HaCaT cells

RAGE acts as a key factor in transmitting AGEs and other associated signaling pathways^27^. Our obtained results showed that GO-AGEs treatment upregulated RAGE protein expression in HaCaT cells compared to that in the control group. However, RAGE expression was 1.5 times higher in the GO-AGEs + UVB treatment group than in the UVB and GO-AGEs treatment groups. Similarly, COX2 protein expression was enhanced approximately two-fold in the GO-AGEs + UVB treatment group. Besides that, the phosphorylation of P65 (NF-kB) was enhanced in favor of the expression of inflammatory cytokine proteins (Fig. 2A–E). Moreover, the GO-AGEs + UVB treatment group was observed to initiate the phosphorylation of P38 by approximately 1.5 times when compared to that in the control and UVB groups (SF 1C and SF 1D).

### GO-AGEs induce the oxidative stress along with the upregulatory expression of several inflammatory genes in NHEK cells

**Figure 3:**
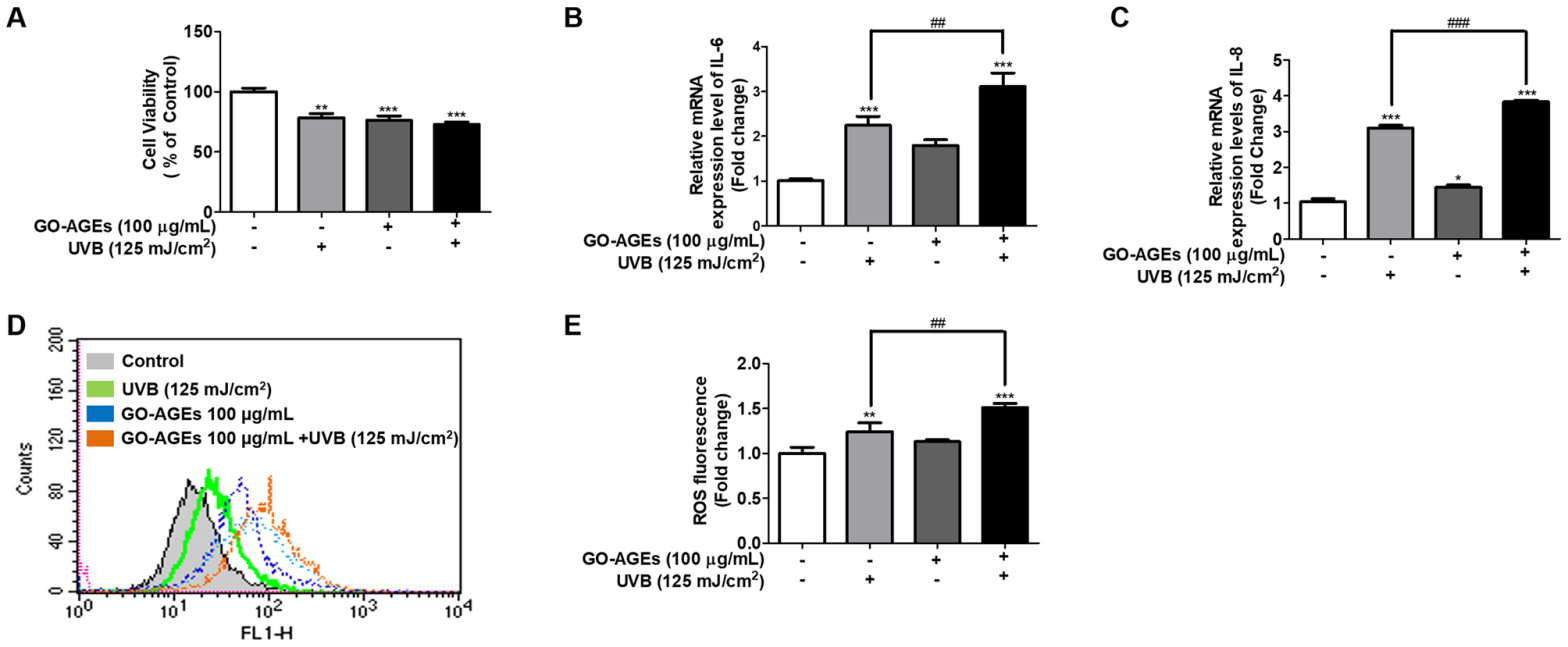
GO-AGEs induce oxidative stress along with the up-regulatory expression of several inflammatory genes in NHEK cells. (**A**) MTT assay showing the cytotoxic effects of GO-AGEs (100 μg/mL) in the presence or absence of UVB irradiation (125 mJ/cm^2^) along with the control group in NHEK cells. (**B**,**C**) qRT-PCR results showing the upregulation of the relative mRNA expression levels of IL-6 (**B**) and IL-8 (**C**) genes upon treatment with GO-AGEs (100 μg/mL) in the presence or absence of UVB irradiation (125 mJ/cm^2^). (**D**) FACS analysis showing the oxidative stress upon treatment with the with GO-AGEs (100 μg/mL) in the presence or absence of UVB irradiation (125 mJ/cm^2^). (**E**) The fold change of ROS level in NHEK cells. Each value represents the mean ± SEM of triplicate experiments. *p < 0.05, **p < 0.01, and ***p < 0.001 vs. the control group and ^##^p < 0.01, and ^###^p < 0.001 vs. the UVB treatment group induced by UVB irradiation (125 mJ/cm^2^).

In NHEK cells, GO-AGEs upregulated the expression of different inflammatory genes (IL-6 and IL-8). Approximately three fold increase in both IL-6 and IL-8 mRNA expression levels was observed upon individual treatment with GO-AGEs and GO-AGEs + UVB group. However, UVB treatment alone increased the expression of these genes by approximately two- and half-fold in both cases. In particular, the GO-AGEs + UVB treatment groups showed more than a three-fold increase in IL-6 and IL-8 mRNA levels compared to that in the other groups (Fig. 3B,C). In addition, FACS analysis confirmed that all of their individual treatments (GO-AGEs, UVB, and GO-AGEs + UVB) increased the formation of ROS to induce oxidative stress. More specifically, the GO-AGEs + UVB group significantly induced the formation of ROS around 1.5-fold in comparison to that in the other groups (Fig. 3D–E).

### GO-AGEs enhance the degradation of the extracellular matrix by increasing matrix metalloproteinase release and decreasing the expression of COL1A and SIRT1 in NHDF cells

**Figure 4:**
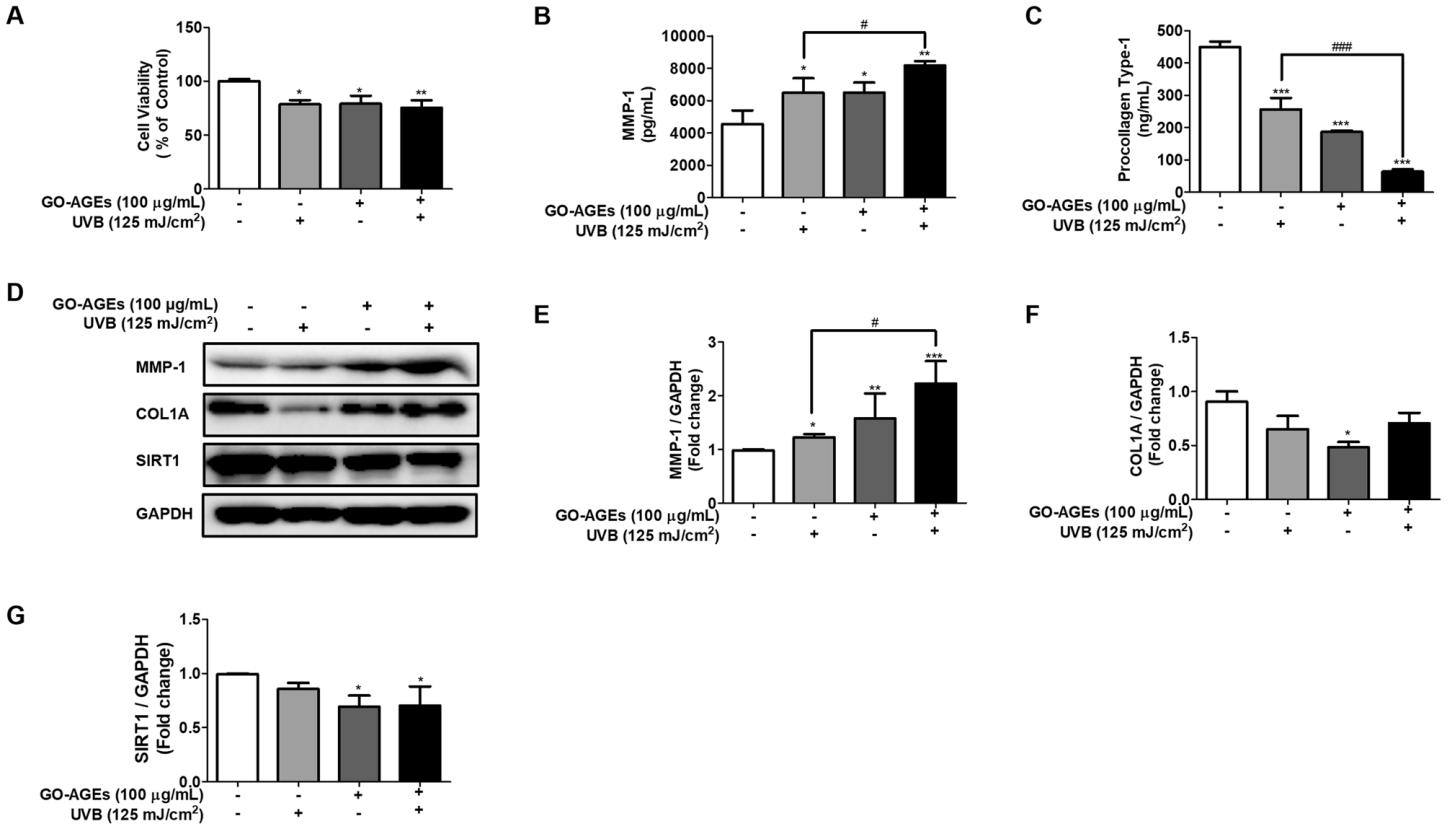
GO-AGEs enhance the degradation of extracellular matrix by increasing the matrix metalloproteinase release while decreasing expression of COL1A and SIRT1 in NHDF cells. (**A**) MTT assay showing cytotoxic effects of GO-AGEs (100 μg/mL) in the presence or absence of UVB irradiation (125 mJ/cm^2^), along with the control group in NHDF cells. (**B**,**C**) ELISA results demonstrating the MMP-1 (**B**) and COL1A (**C**) levels in the conditioned medium of NHDF cells upon treatment with GO-AGEs (100 μg/mL) in the presence or absence of UVB irradiation (125 mJ/cm^2^) along with the control group. (**D**) Western blot analysis showing the expression level of MMP-1, COL1A, and SIRT1 proteins after treatment with GO-AGEs (100 μg/mL) in the presence or absence of UVB irradiation (125 mJ/cm^2^) along with the control group. (**E**–**G**) Quantification of the expression levels of MMP-1 (**E**), COL1A (**F**), and SIRT1 (**G**). Each value represents the mean ± SEM of triplicate experiments. *p < 0.05, **p < 0.01, and ***p < 0.001 vs. the control group and ^#^p < 0.05 and ^###^p < 0.001 vs. the UVB treatment group induced by UVB irradiation (125 mJ/cm^2^).

According to our results as shown in Fig. 4B, GO-AGEs treatment particularly the GO-AGEs + UVB treatment group elevated the release of MMP1 in the conditioned media of NHDF cells. In addition, the GO-AGEs + UVB treatment group enhanced its expression at the protein level by approximately two-fold compared to that in the control and UVB treatment groups. Regarding collagen degradation, COL1A levels decreased significantly upon treatment with GO-AGEs + UVB (Fig. 4B,C). However, at the protein level, UVB treatment remarkably degrade COL1A compared with that in the other groups. Additionally, the downregulated expression of SIRT1 was notable in the GO-AGEs group, and more specifically, in the GO-AGEs + UVB treatment group compared to that in the individual groups of UVB and GO-AGEs (Fig. 4D–G).

### Molecular docking analysis confirms the binding affinities for all of its target proteins

**Figure 5:**
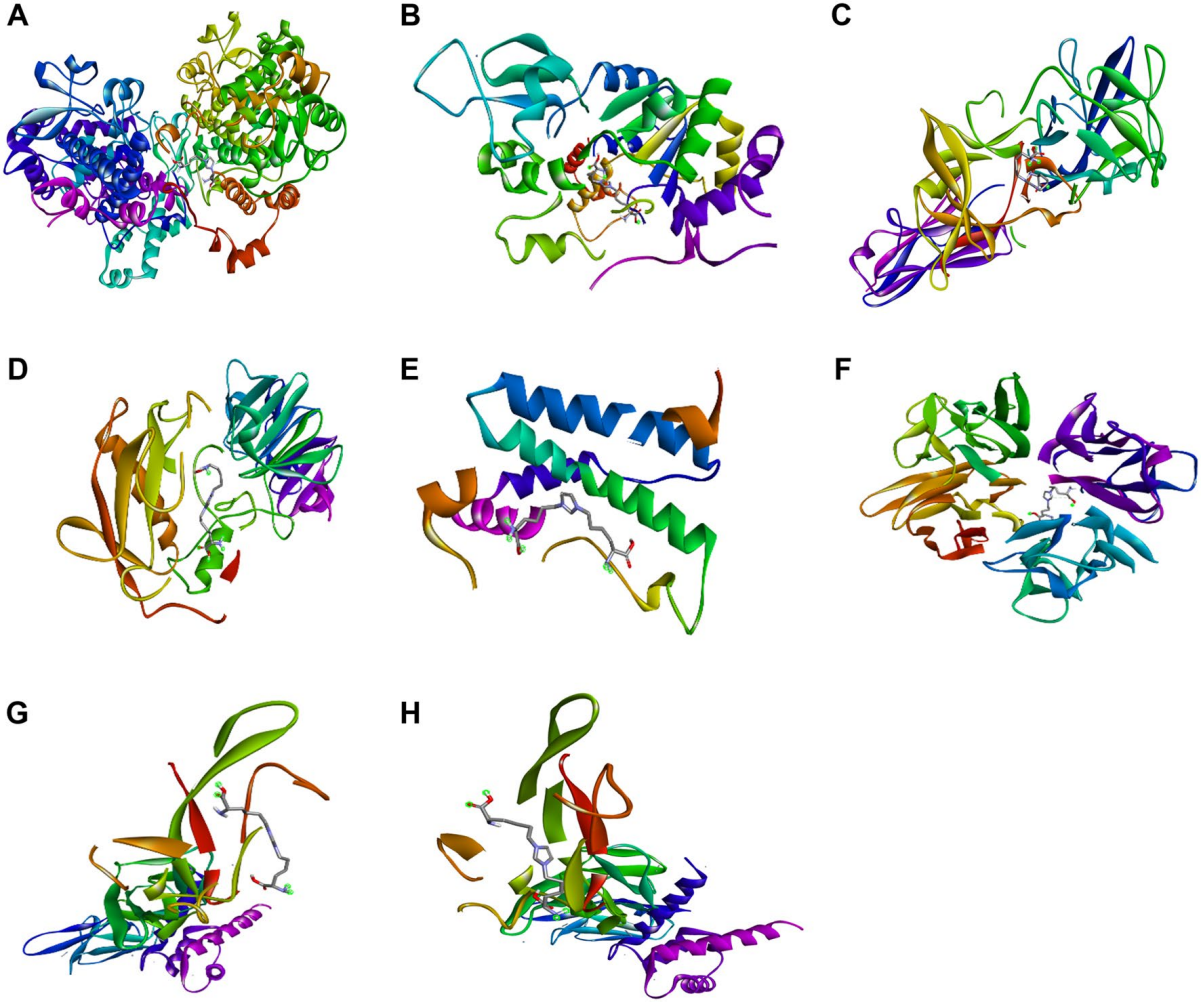
Molecular docking analysis of glyoxal-lysine dimer (GOLD) with different inflammatory proteins performed by the AutoDock Vina program. The complex structures of GOLD with COX2 (**A**), SIRT1 (**B**), IL-1β (**C**), MMP-1 (**D**), P65 (**E**), IL-6 (**F**), TNF-α (**G**), and RAGE (**H**).

**Figure 6:**
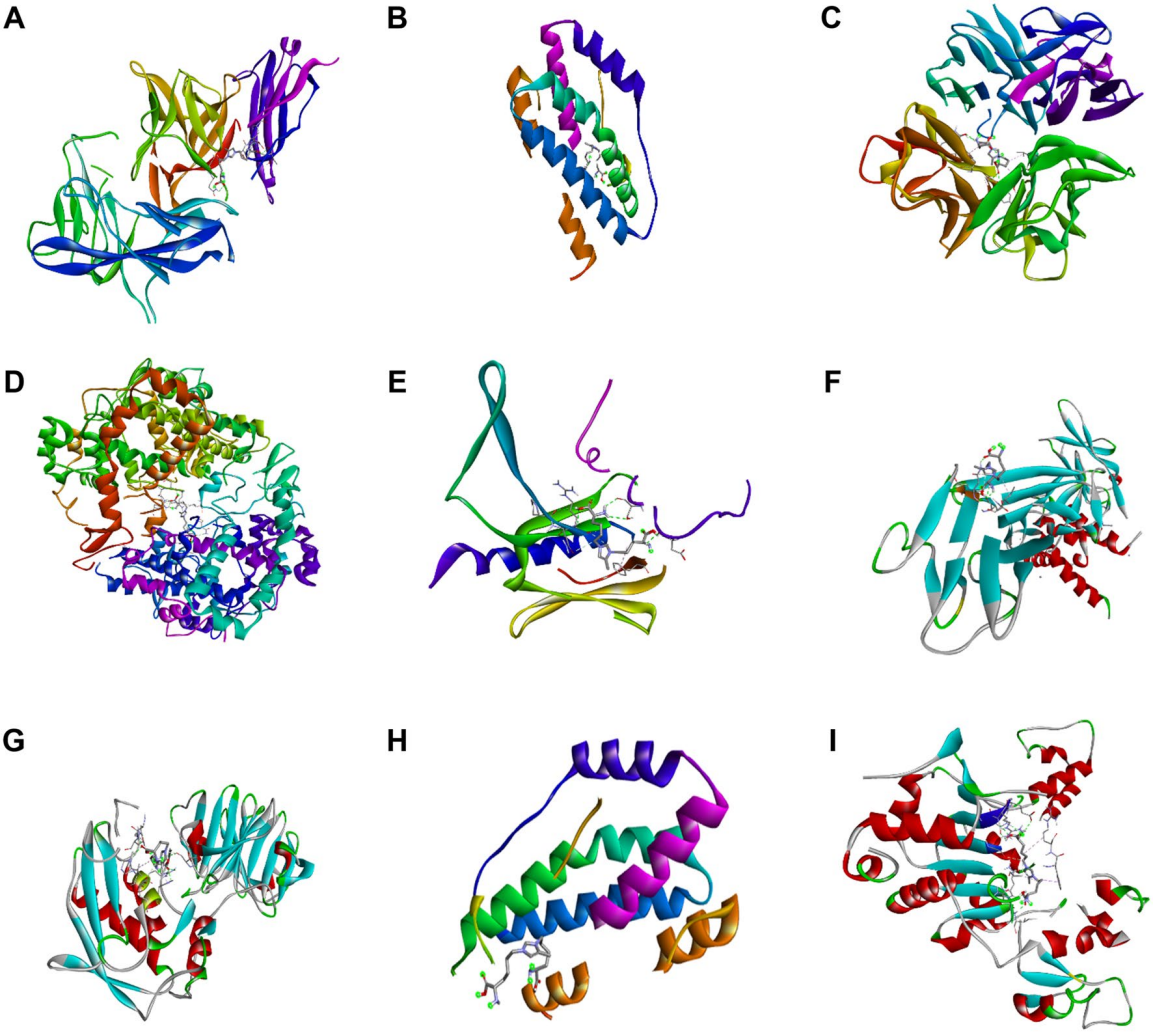
Molecular docking analysis of methylglyoxal-lysine dimer (MOLD) with different target proteins performed by the AutoDock Vina program. The complex structures of MOLD with IL-1β (**A**), IL-6 (**B**), TNF-α (**C**), COX2 (**D**), P65 (**E**), RAGE (**F**), MMP-1 (**G**), COL1A (**H**), SIRT1 (**I**).

Molecular docking study evaluated the structural interactions and binding of the GOLD, with RAGE and other structural and inflammatory target proteins in skin. The outcomes of the molecular docking study, binding scores and chemical bonds involved in binding are listed in Table 1 and Fig. 5. GOLD has shown the strongest binding scores towards all of the target proteins: COX2 (− 6.7 kcal/mol), SIRT1 (− 6.6 kcal/mol), IL-1β (− 5.9 kcal/mol), MMP1-1 (− 5.9 kcal/mol), P65 (− 5.4 kcal/mol), IL-6 (− 4.5 kcal/mol), TNF-α (− 4.5 kcal/mol), RAGE (− 4.8 kcal/mol), and COL1A (− 4.5 kcal/mol). GOLD has shown the highest binding affinities towards COX2 (− 6.7 kcal/mol) with hydrogen bonding at GLY(A:263), ASP(A:272), GLN(A:345), ASN(A:346), ILE(A:347), GLY(A:440), and SER(A:441), along with other chemical bonds. Although its binding affinities towards IL-6, TNF-α, and COL1A were lower (− 4.5 kcal/mol) compared to others, this score is evident enough for GOLD to interact and transmit signals for the initiation of different inflammatory signaling pathways (SF 1, SF 2, and SF 3). To obtain a comparative overview of GOLD and MOLD as potential inducers, molecular docking of MOLD against inflammatory and membrane structural target proteins was evaluated. According to the obtained binding scores and different bond positions, MOLD showed strong binding affinity towards all target proteins, followed by GOLD, except a few instances, (as demonstrated in Fig. 6, SF 4, and SF 5). MOLD demonstrated the highest affinity towards COX2 (− 6.9 kcal/mol) via hydrogen bonds (GLN A:374,ARG A:376,ASN A:375,ASN B:537,ASN B:375, and GLY B:536) and other bonds. In contrast, the lowest scores were noticed in case of MOLD against COL1A (− 4.1 kcal/mol) and RAGE (− 4.4 kcal/mol). Both GOLD and MOLD showed significant interactions with all the target proteins in initiating the signal transduction of skin inflammation, as concluded through these molecular simulations.

**Table 1:** List of docking scores of the complex form of GOLD with different proteins.

Proteins	Ligands	Binding affinity (kcal/mol)	H-bond position
IL-1β (PDB ID:1ITB)	GOLD	− 5.9	LYS A:27, SER A:21, ARG B:9
	MOLD	− 5.5	GLN A:149, ASP B:239, SER B:238, THR B:207, HIS B:301
IL-6 (PDB ID:1ALU)	GOLD	− 4.5	LYS A:171, ARG A:179, GLN A:175
	MOLD	− 4.5	SER A:176, ARG A:179, SO A: 4290, SO A: 4291
TNF-α (PDB ID:2AZ5)	GOLD	− 5.5	TYR D:151, LEU D:120, SER D:60, LEU C:120, SER C:60, TYR C:151
	MOLD	− 5.3	TYR B:151, GLY A:121, TYR A: 151, GLN A:61
COX2 (PDB ID:5KIR)	GOLD	− 6.7	GLY(A:263), ASP(A:272), GLN(A:345), SN(A:346), LE(A:347), GLY(A:440), SER(A:441)
	MOLD	− 6.9	GLN A:374, ARG A:376, ASN A:375, ASN B: 537, ASN B:375, GLY B:536
P65 (PDB ID:5URN)	GOLD	− 5.4	SER B:535, ARG A:86, ASP B:531
	MOLD	− 5.3	ASP B:531, ARG A:86, PRO A:84, SER B:535
RAGE (PDB ID:4YBH)	GOLD	− 4.8	VAL A:78, LEU A:49
	MOLD	− 4.4	GLU A:94, GLY A:56, ASN A:54
MMP-1 (PDB ID:1FBL)	GOLD	− 5.9	SER A:227, HIS A:222, GLU A:219, ALA A:182
	MOLD	− 6.1	ARG A:214, HIS A:218, PRO A:238, ASN A:180, HIS A:183, GLU A:219
COL-1A (PDB ID.: 1BVK)	GOLD	− 4.5	GLY B:45, ARG A:14, ALA B:43, THR B:41
	MOLD	− 4.1	ARG B:44, GLY B:45, GLY C:78, ALA C:77
SIRT1 (PDB ID:4ZZJ)	GOLD	− 6.6	ASN A:346, ILE A:347, GLN A:345, ASP A:272
	MOLD	− 6.6	GLN A:345, ILE A:347, ASN A:346, GLY A:440, ASN A:465, GLU A:467, ARG A:466

## Discussion

AGEs have gained considerable attention now-a-days because of their pivotal roles in several diseases, including skin aging^28^. As a result of glycation and oxidation, AGEs formed and accumulate in various organs, including in the skin, especially in the epidermal and dermal layers^29^. Previous studies have shown that endogenously-produced dicarbonyl substances such as glyoxyl (GO), methylglyoxyl (MGO), and carboxymethyl-lysine (CML) serve as potential precursors for AGEs formation^30^. AGEs have been confirmed to be synthesized from exogenous sources such as food, UVB irradiation, and smoking, among others^31,32^. Dicarbonyl stress by MGO is a dysfunction state where MGO or GO accumulate as consequence of their decreased activity of the detoxifying systems. Accumulation of MGO in skin tissues leads to structural and functional changes. MGO, GO and its derived advanced glycation end products (AGEs) have been reported to cause inflammation-induced skin aging, psoriasis, and dermatitis^33^. Till now, there are many reports on the biological function of MGO on the skin disease, but relatively few reports on the action of GO. But GO is also highly reactive dicarbonyl compound that has been reported to produce CML, a well-known biomarker of glycation; thus GO and its derived AGEs are the best glycating agents for conducting in vitro glycation studies^21^. However, most studies have focused on MGO or 3-DG to synthesize and analyze the in vitro formation of AGEs^34^. Less attention has been paid to GO, particularly GO-derived AGEs (GO-AGEs), despite their significant implications in several inflammatory disorders^22^. To date, few studies have been conducted on GO-AGEs, most related to their structural identification and rarely in terms of skin inflammaging. This research gap has ignited to perform this study for the first time focused on investigating the effects of GO-AGEs on skin inflammaging and revealing its molecular mechanism.

Based on our preliminary study results, in HaCaT cells, GO-AGEs at 100 µg/mL were eligible enough to induce the secretion of IL-1β. However, this result was more promising when GO-AGEs were irradiated with UVB (125 mJ/cm^2^), as shown in Supplementary Fig (SF 1B). However, the cytotoxicity was significantly different in the GO-AGEs + UVB treatment group (GO-AGEs at 100 µg/mL combined with UVB irradiation at 125 mJ/cm^2^) among other concentrations of GO-AGEs (50, 100, and 250 µg/mL) and the control group, as shown in Supplementary Figure (SF 1A). The GO-AGEs group at 250 µg/mL were excluded from further study due to its higher cytotoxicity. Therefore, subsequent studies were conducted with the GO-AGEs group alone and in the presence or absence of UVB irradiation to reveal the molecular mechanism of inflammation induced skin aging called inflammaging.

Skin inflammaging refers to continuous, low level inflammation associated with aging which gradually causes skin tissue damage. Therefore, skin inflammaging is accompanied by substantial rise in different inflammatory cytokines and chemokines (IL-1β, IL-6, TNF-α, and IL-8, etc.) in response to numerous stimuli, such as UVB, and endogenous substances, among others^35^. Reportedly, these cytokines ( IL-6, IL-1β, and TNF-α, etc.) are well studied in terms of their relationship to inflammatory disorders, in addition to skin inflammaging^36^. According to our study results, individual treatment of the GO-AGEs, and UVB group, has been noted to significantly induce the release of different pro-inflammatory cytokines (IL-1β, IL-6, and TNF-α) when compared to that in the control groups in HaCaT cells. Interestingly, GO-AGEs + UVB treatment group, was observed to induce the release of these proinflammatory cytokines(IL-1β, IL-6, and TNF-α) potentially more than the GO-AGEs and UVB treatment groups, as shown in Fig. 1B–D. In particular, IL-1β was found to increase dramatically via the GO-AGEs + UVB treatment group in a comparison to GO-AGEs or UVB groups separately (Fig. 1B), whereas IL-6 (Fig. 1C) and TNF-α (Fig. 1D) were also seen to follow a similar pattern of incremental changes in all the above-mentioned groups. Additionally, GO-AGEs + UVB treatment induced the release of NO significantly compared to that in the other groups (Fig. 1E). However, cell viability was notably distinguishable among the GO-AGEs, UVB, and GO-AGEs + UVB treatment groups in comparison to that in the control group (Fig. 1A). Moreover, NHEK cells treated with GO-AGEs + UVB group were reported to enhance ROS release, which is responsible for potentially creating 1.5-times the amount of oxidative stress compared to the control and other groups (Fig. 3D,E). The upregulated expression of IL-6 and IL-8 at the mRNA level was seen in GO-AGEs treatment group, which was augmented more sharply upon combination with UVB irradiation (Fig. 3B,C). However, the cell viability rate was noted to decrease following a similar identical pattern in all the individual treatment groups of UVB, GO-AGEs, and GO-AGEs + UVB (Figs. 1A, 3A).

The AGEs-RAGE interaction may be a potential mechanism underlying the accelerated skin inflammaging. AGEs bind to RAGE to initiate different downstream inflammatory signaling pathways (NF-κB, COX2, etc.), which initiate inflammatory responses through a cascade of proinflammatory cytokines and chemokines^37^. Our data demonstrated that the treatment of UVB and GO-AGEs separately and significantly activated RAGE protein expression estimated one- and half-fold, which was further enhanced by GO-AGEs + UVB treatment (Fig. 2A,B). The expression of IL-1β was enhanced remarkably upon treatment with UVB, GO-AGEs and GO-AGEs + UVB groups (as shown in Fig. 2A,C). However, more than threefold expression of IL-1β protein was seen with the GO-AGEs + UVB treatment group. Additionally, the phosphorylation of P65 was noted as being slightly increased in all three of the above mentioned groups when compared to that of the control group (Fig. 2A,E). NO synthetase (NOS) and COX-2 have been confirmed to release NO and prostaglandins in response to inflammatory stimuli that lead to chronic diseases, including aging^38,39^. Based on our data, the expression of COX2 was noted to increase approximately two-fold in the GO-AGEs + UVB treatment group in comparison to the control and UVB treatment groups (Fig. 2A,D), it was previously able to release NO in the conditioned medium when compared to that in the other groups (Fig. 1E). Collectively, the above findings demonstrate that GO-AGEs in the presence of UVB were able to significantly activate AGEs-RAGE signaling along with several other inflammatory proteins to induce skin inflammaging.

Based on the molecular mechanism of skin aging, AGEs inhibit collagen synthesis and degradation in dermal fibroblasts upon activating the AGE-RAGE signaling pathway. Activated MMPs may degrade collagen, elastin and other ECM, probably resulting in skin aging. Especially skin collagen fiber firstly cleaved by MMP-1 and then MMP-2 diffusing across them which cause unwinding of the fibrils. Furthermore, AGEs increase the synthesis of matrix metalloproteinases (MMPs), and trigger apoptosis. Our study’s findings agree with previous studies showing that AGEs slightly inhibited the synthesis of collagen 1A (Fig. 4D,F) in NHDF cells. However, COL1A was significantly degraded upon treatment with GO-AGEs + UVB when compared to that the control and UVB groups (Fig. 4C). Additionally, MMP-1 expression was increased approximately two-fold in the protein level by the GO-AGEs group alone and in the GO-AGEs + UVB treatment group (Fig. 4D,E). Similarly, its secretion into the cell supernatant was higher in the GO-AGEs + UVB group than that in the other groups (Fig. 4B). These results imply that an increase of MMP1 expression assists in collagen degradation and facilitates extracellular matrix degradation. MMPs regulate the important functions related to skin inflammation including activity of inflammatory cytokines and chemokines. The role of sirtuins in inflammation and aging has already been confirmed in several studies and their deletion or deactivation can increase inflammation^40^. According to our results, GO-AGEs, individually and in combination with UVB irradiation, acted as deactivators of SIRT1 (Fig. 4D,G), indicating its strong association with inflammation induced skin aging. Taking together, GO-derived AGEs (GO-AGEs) in the presence of UVB can act as potential inducer of inflammaging and have significant implications for the degradation of extracellular matrix components.

According to several in-vitro and in-vivo studies, UVB irradiation promotes AGEs production which further interacts with RAGE and activates several inflammatory signaling pathways (NF-B, MAPK, etc.) to enhance the release of inflammatory cytokines. UVB irradiation enhances the breakdown of the extracellular matrix, especially that of collagen, through the upregulation of MMP-1 to induce inflammaging^41^. In HaCaT cells, the individual treatment of UVB and GO-AGEs has demonstrated significant increase in the release of IL-1β, IL6, TNF-α, and NO in the cell supernatant following a two-fold increment compared to that in the control group. However, when GO-AGEs were combined with UVB, the GO-AGEs + UVB treatment group showed dramatically higher cell viability than in the UVB and GO-AGEs groups individually (Fig. 1). Subsequently, upon treatment with the GO-AGEs + UVB group, the expression of COX2 and IL-1β protein was remarkably augmented by around two- and three-fold, respectively, among other groups. Furthermore, it upregulated the expression of RAGE protein along with the phosphorylation of P65 to activate inflammatory signaling pathways (Fig. 2). Following a similar trend, it was observed to create the oxidative stress through ROS regulation (approximately one- and half-fold) along with the upregulation of IL-6 and IL-8 at the mRNA level in NHEK cells (Fig. 3). In NHDF cells, the level of MMP1 in the conditioned media and the protein level increased significantly after treatment with GO-AGEs + UVB. In addition, the GO-AGEs + UVB treatment group showed a three-fold decrease in Procollagen Type-1 compared to that in the control group and a half- fold decrease compared to the UVB and GO-AGEs treatment groups. Similarly, Collagen type 1 protein was downregulated (Fig. 4). In a comparative evaluation of GO-AGEs and UVB as stimulants or inducers of skin inflammaging, GO-AGEs demonstrated potential implications compared to UVB irradiation. Precisely, GO-AGEs initiated several crucial signaling cascades more promptly than UVB irradiation, however, the result was more prominent when GO-AGEs were combined with UVB irradiation.

To date, molecular and homological docking, molecular dynamics, and simulation studies have been widely used as scientific tools to predict and understand the mechanisms of biological processes due to their economic and time-saving benefits. Previous studies identified GOLD as a novel ligand for molecular docking experiments against multiple targets^42^. From our obtained results, GOLD has shown the highest docking score towards COX2 and SIRT1 around − 6.7 kcal/mol and − 6.6 kcal/mo,l respectively with the formation of different types of chemical bonds at multiple positions. In addition, the binding affinities and different bonding of GOLD towards all its target proteins were noted to activate several signaling pathways involved in inflammaging (Table 1 and Fig. 5). The molecular docking study of MOLD^32^ against all target proteins was also performed to evaluate the comparative potentiality of GOLD and MOLD as ligands. Based on the obtained scores and H-bonding at different positions, MOLD showed its promising potentiality as a significant inducer of skin inflammation, which can be evaluated in further studies. In order to evaluate the comparative potentiality of GOLD and MOLD as ligands, separate molecular docking study of MOLD^32^ against all of the above mentioned target proteins was also investigated. According to the obtained scores and H-bonding at different positions, MOLD has promising potentiality as a strong inducer of skin inflammation, which can be evaluated in further studies. The obtained results recommended that GOLD and MOLD exhibited different modes of interaction, with GOLD having higher binding affinities (kcal/mol) for the proteins IL-1β (− 5.9), TNF-α (− 5.5), RAGE (− 4.8), COL 1A (− 4.4), and MOLD having higher affinities for COX2 (− 6.7) and MMP-1 (− 6.1). Therefore, GOLD, one of GO derived AGEs, may be a potential inducer of skin inflammaging. Based on these findings, a further study comparing the difference and commonalities by treating the GO-AGEs, MGO-AGEs, GOLD and MOLD under same conditions in the UVB-induced skin inflammaging will be commenced in future.

**Figure 7:**
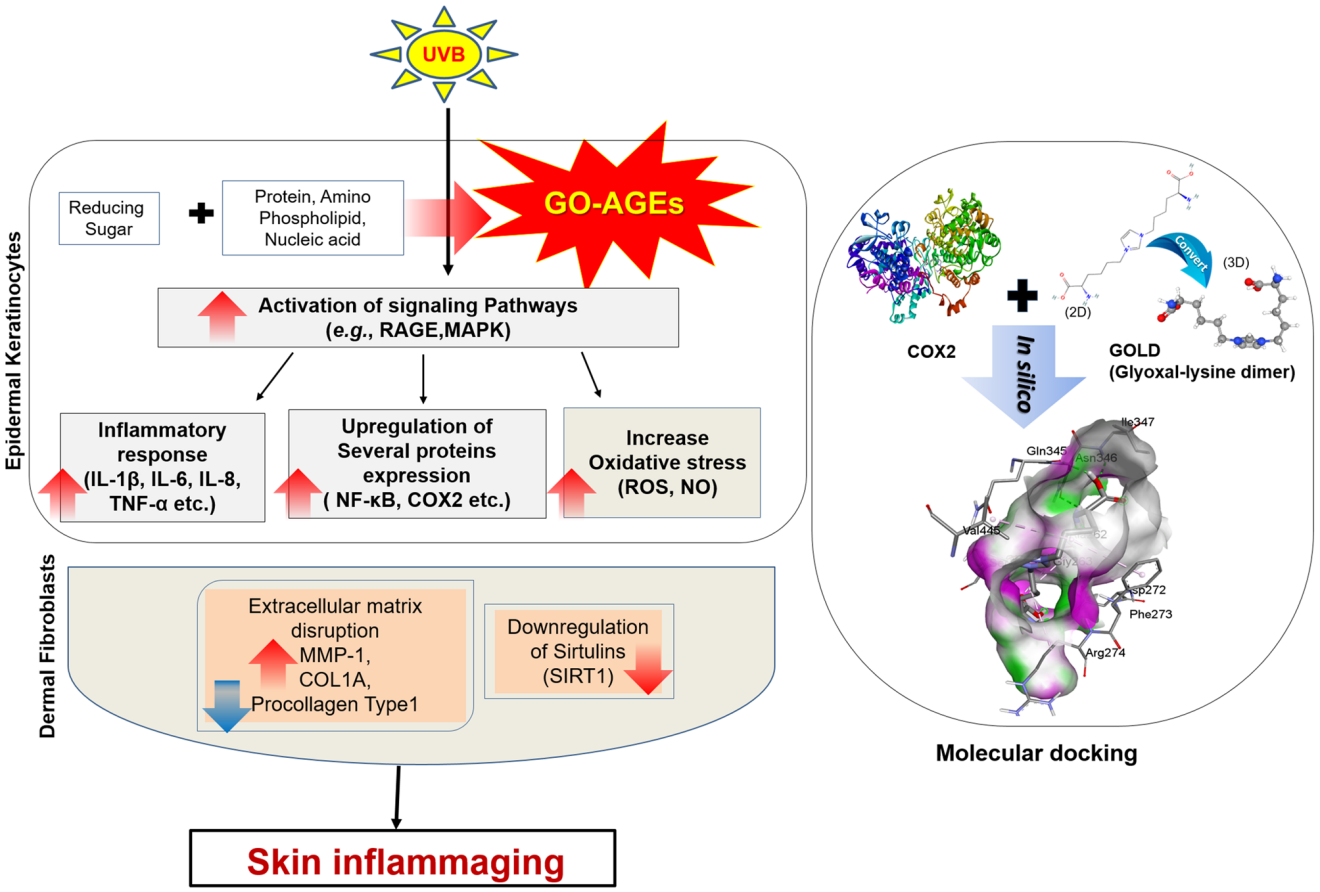
Proposed mechanism for skin inflammaging effect of GO-AGEs.

In summary, this study sheds light on the potentiality of GO-AGEs on skin inflammaging through its applications on epidermal (keratinocytes) and dermal (fibroblasts) layers as presented the graphical abstract in Fig. 7. In NHEK cells, GO-AGEs in the presence of UVB significantly induced oxidative stress via ROS release, and upregulation of IL-6 and IL-8 genes. Similarly, GO-AGEs in the presence of UVB were observed to increase the release of IL-6, IL-1β, and TNF-α, potentially with further activation of RAGE and its downstream signaling pathways in favor of the activation of skin inflammaging in HaCaT cells. In addition, a significant increase in collagen degradation and the release of matrix metalloproteinases were noted, along with the deactivation of SIRT1 in NHDF cells. Therefore, it can be strongly stated that GO-AGEs alone, or in combination with UVB, can act as a potential inducer of skin inflammaging. Skin inflammaging by GO-AGEs can be defined as an age-related increase in the levels of proinflammatory markers in UVB-irradiated skin, and are, thus, novel anti-skin aging therapeutic targets. This study has focused on the implications of GO-AGEs individually and in combination with UVB in the context of skin inflammaging along with revealing its molecular mechanism, which needs further validation through in vivo deep study and by incorporating other cell lines.

## Materials and methods

### Chemicals and reagents

Dulbecco’s modified Eagle’s medium (DMEM), fetal bovine serum (FBS), dimethylsulfoxide (DMSO), penicillin, and streptomycin were obtained from Gibco-BRL (Grand Island, NY, USA). Glyoxal solution (GO), sodium azide, and 3-(4,5-dimethylthiazol-2-yl)-2,5-diphenylter-thiazolium bromide (MTT) were obtained from Sigma (St. Louis, MO, USA). ELISA kits (IL-6 and TNF-α) were from R&D Systems Inc. (Minneapolis, MN, USA). PRO-PREP™ protein extraction solution, and enhanced chemiluminescence (ECL) detection kits were availed from Intron (Sungnam, Korea). Antibodies against GAPDH, RAGE, IL 1 β, COX2, SIRT1, MMP-1, Type I Procollagen (COL1A), P38, phospho-P38 (p-P38), P65, phospho-P65 (p-P65), and secondary antibodies conjugated to horseradish peroxidase were purchased from Santa Cruz (Dallas, TX, USA), Cell Science (Canton, MA, USA), and Cell Signaling Technology (Beverly, MA, USA).

### Preparation of GO-AGEs and treatment of cells

GO-AGEs were prepared^43^ by incubating bovine serum albumin (10 mg/mL), 0.2% sodium azide with 10 mM glyoxal in PBS (pH 7.4), at 37 °C in the dark for 7 days. After 7 days, the samples were evaporated, dialyzed, filtered through desalting columns, and stored in freeze dried condition until further use. The stock solution of GO-AGEs was prepared in serum-free media, which was further diluted to 100 µg/mL and used immediately to treat the cells.

### Cell culture

Normal human dermal fibroblast (NHDF) cells (passage numbers between 5 and 10) and immortal keratinocytes (HaCaT cells) were obtained from the Korean Cell Line Bank (Seoul National University, Seoul, Korea). Both cell lines were cultured in high glucose DMEM (Thermo Scientific, Waltham, MA) with 10% FBS and 1% penicillin–streptomycin (PS). Cultured cells were maintained in a humidifier at 37 °C and 5% CO_2_. UVB irradiation was performed following a previously reported method with slight modification^44^ at UVB (125 mJ/cm^2^) using a UVB irradiation machine (Bio-Link BLX-312; Vilber Lourmat GmbH, Marne-la-Vallée, France).

### Cell viability assay

For the MTT assay, HaCaT (3 × 10^4^ cells /well) and NHEK cells (1 × 10^4^ cells /well) were seeded into 96-well plates, whereas NHDF (3 × 10^3^ cells/well) were seeded into 48 well plates, which were further incubated at 37 °C in 5% CO_2_ for 24 h. For UVB irradiation, all cell lines (HaCaT, NHEK, and NHDF cells) were suspended in a small amount of PBS and then exposed to UVB (125 mJ/cm^2^), followed by three washings with warm PBS. Then, the culture media was removed, and 0.5 mg/mL of the MTT solution was added after 24 h and 48 h of incubation. After 1 h, the MTT solution was removed, and 100 µL of DMSO was added to each well. The absorbance was measured at 570 nm using a microplate reader (Molecular Devices E09090; San Francisco, CA, USA).

### Measurement of pro-inflammatory cytokines (IL-1β, IL-6, and TNF-α) levels by enzyme-linked immunosorbent assay (ELISA) in HaCaT cells

HaCaT cells were seeded into 96-well plates (3 × 10^4^ cells/well) for 24 h and afterwards treated with GO-AGEs (100 µg/mL) in the presence or absence of UVB irradiation (125 mJ/cm^2^) separately. After 24 h incubation, the supernatant was collected to check the levels of IL-1β, IL-6, and TNF-α by ELISA following the manufacturers’ instructions.

### Measurement of MMP-1 and COL1A in NHDF cells

NHDF (3 × 10^3^ cells/well) were seeded into 48-well plates for 24 h, after which the cells were treated with GO-AGEs (100 µg/mL) in the presence or absence of UVB irradiation (125 mJ/cm^2^). After 48 h of incubation, the MMP-1 and COL1A concentrations were analyzed using ELISA kits (Human Total MMP-1; R&D Systems Inc.; COL1A C-Peptide ELISA Kit, Takara).

### Measurement of ROS Production

NHEK (3 × 10^3^ cells/well) cells were seeded into 48-well plates for 24 h at 37 °C in 5% CO_2_ following the treatment with GO-AGEs (100 µg/mL) in the presence or absence of UVB irradiation (125 mJ/cm^2^). Furthermore, the cells were incubated for 24 h and stained with 30 μM 2′,7′-dichlorofluorescein diacetate (DCFH-DA; Sigma-Aldrich) for 30 min at 37 °C in a CO_2_ incubator. After that, cells were analyzed using FACS (FACS Calibur™; Becton-Dickinson, San Jose, CA, USA).

### Determination of nitric oxide (NO) production

HaCaT cells (3 × 10^4^ cells/well) were seeded for 24 h at 37 °C in 5% CO_2_. After 24 h OF incubation, the cells were treated with GO-AGEs (100 µg/mL) in the presence or absence of UVB irradiation (125 mJ/cm^2^) separately and kept for 24 h of further incubation. The accumulated quantity of nitrite in the culture medium was measured as an indicator of NO production following a previously-described method^45^. Briefly, 50 µL of cell culture medium was mixed with 50 µL of Griess reagent (1% sulfanilamide and 0.1% naphthyl ethylene diamine dihydrochloride in 2.5% phosphoric acid) and kept for 10 min incubation period at room temperature. The absorbance was measured at 540 nm using a microplate reader.

### Quantitative real time-reverse transcription-polymerase chain reaction (qRT-PCR)

Total RNA from NHEK cells (6-well plates, 3 × 10^5^ cells/well) was isolated using an RNA extraction kit (KeyGEN BioTECH, Nanjing, China). cDNA was synthesized from total RNA, and qRT-PCR was performed using the PrimeScript RT reagent kit (Takara, Beijing, China), according to the manufacturer’s instructions. The primer sequences used for qRT-PCR were as follows: IL-6, forward: CACCGGGAACGAAAGAGAAG and reverse: TCATAGCTGGGCTCCTGGAG; IL-8, forward: TAAAGACATACTCCAAACCTTTCCAC and reverse: AAGCTTTACAATAATTTCTGTGTTGGC; and GAPDH, forward: TCCACTGGCGTCTT CACC, reverse: GGCAGAGATGATGACCCTTTT. The mRNA expression levels of IL-6 and IL-8 Mrna was normalized to those of GAPDH.

### Western blotting

HaCaT cells were harvested, washed with cold PBS (1 ×), and lysed in PRO-PREP™, containing protease and phosphatase inhibitors. The obtained protein content was estimated using the Bradford assay. 30 μg of protein from each group were loaded and separated by 6–10% SDS-PAGE gel electrophoresis, then transferred to nitrocellulose filter membrane, followed by blocking with 5% skim milk. After transferring, each nitrocellulose membrane containing loaded sample set of control, UVB, GO-AGEs and GO-AGEs + UVB treatment group were further incubated overnight with primary antibodies against GAPDH, RAGE, IL-1β, SIRT1, P65, pP65, P38, pP38, COX2, COL1A, and MMP-1 at 4 °C (three replicates of each protein). The membranes were then incubated with horseradish peroxidase-conjugated secondary antibodies, and protein bands were visualized using enhanced chemiluminescence reagent in the Chemi DocXRS + imaging system (Bio-Rad, Hercules, CA, USA). Finally, densitometric analysis was performed using Image Master TM 17 2D Elite software, version 3.1 (Amersham Pharmacia Biotech, Piscataway, NJ, USA). 

### Molecular docking analysis

Molecular docking studies were performed using AutoDock Vina version 1.1.2 software, following a previously described method^46^. GOLD, a previously reported derivative of GO-AGEs, has been used as a novel AGE ligand to perform molecular simulations to investigate binding affinities against different target proteins^27^. Briefly, GOLD was used as a ligand and the 3D SDF format were attained from PubChem database (https://pubchem.ncbi.nlm.nih.gov/ [accessed on 3rd February, 2023]) of the chemical repository having PubChem CID 46878529. Additionally, the 3D structures of different target proteins were obtained from the Protein Data Bank (PDB) database (https://www.rcsb.org/ [accessed on 3rd February, 2023]) in PDB format. Briefly, PDBQT file containing protein structure with hydrogen atoms in all polar residues was generated, and rotatable ligands were prepared by modifying the existing bonds. Then, protein and ligand fixed docking was performed using the Lamarckian Genetic Algorithm (LGA) method. To create docking site on the target protein, a grid box with the default grid spacing was placed at the center of ligand. The best confirmations with the lowest binding energy were selected, and the interactions between protein–ligand complex conformations were analyzed by hydrogen bonds and bond lengths using Discovery Studio Visualizer 16.1.0.15350. Following the similar procedure as described earlier, methylglyoxal-lysine dimer (MOLD), which is a representative group of MGO-derived AGEs (H. W. Lee et al., 2021b) has been used as ligands (PubChem CID 46878528) in molecular docking against all the target proteins mentioned above.

### Statistical analysis

The results were represented as the mean ± standard error of the mean from three independent experiments. GraphPad Prism 5 (GraphPad Software Inc., La Jolla, CA, USA) was used for statistical analysis. One-way analysis of variance followed by the Newman–Keuls multiple comparison test was performed; *p* < 0.05 was considered statistically significant.

### Supplementary Information

Supplementary Figure 1.

Supplementary Figure 2.

Supplementary Figure 3.

Supplementary Figure 4.

Supplementary Figure 5.

Supplementary Figure 6.

Supplementary Legends.

Supplementary Information.

## Data Availability

All the data generated and/or analyzed during performing this current study are included in this article [and also in its supplementary dataset files]. However, there is no restriction on the availability of materials and data from the corresponding author on reasonable request.
