# Supplementary figures and images for: Glyoxal-derived advanced glycation end products (GO-AGEs) with UVB critically induce skin inflammaging: in vitro and in silico approaches
Source: Sci Rep. 2024 Jan 22;14:1843. doi: 10.1038/s41598-024-52037-z (PMC10800344; doi:10.1038/s41598-024-52037-z)

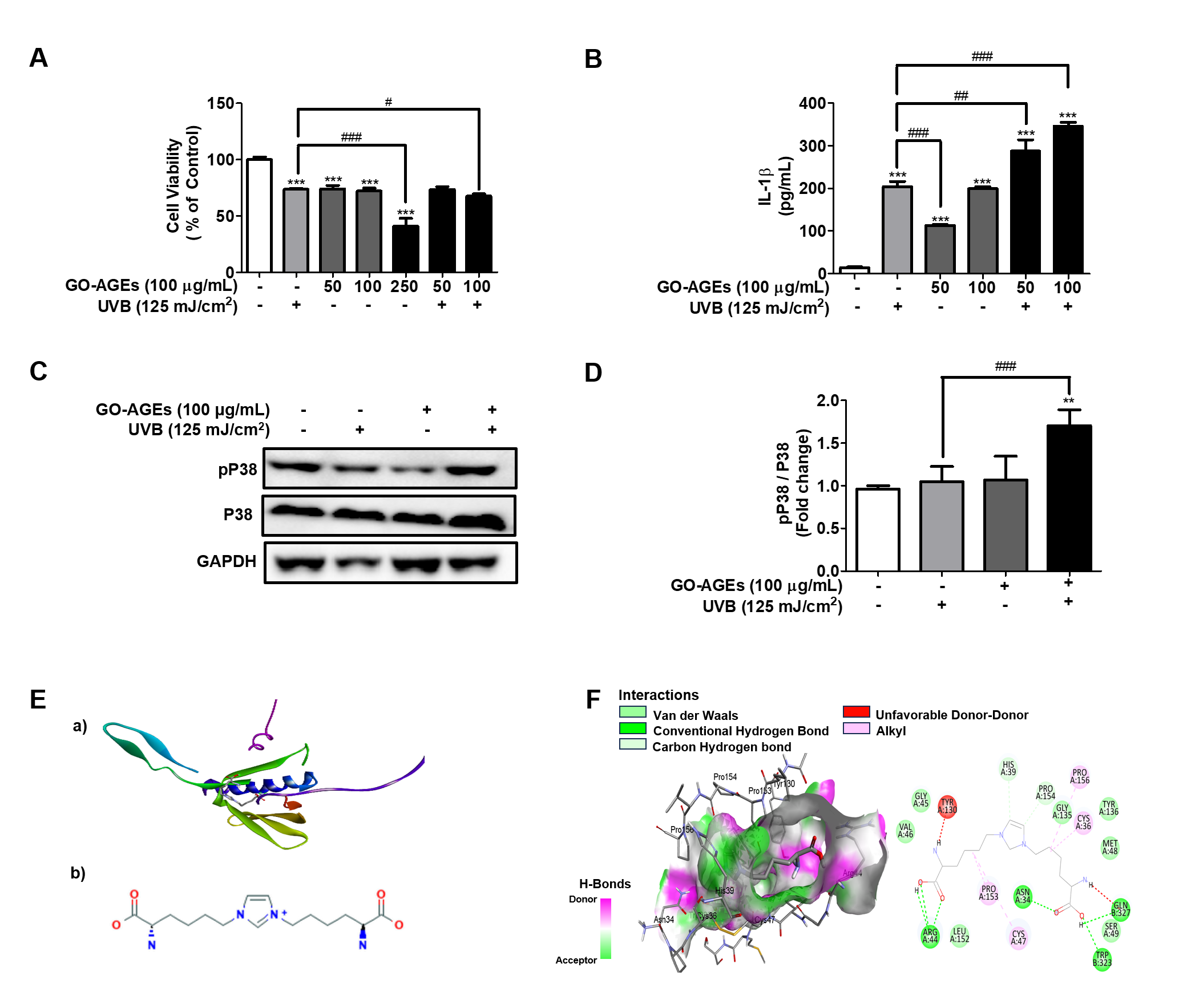

Supplement: Supplementary file 1 — Supplementary Figure 1. [file 41598_2024_52037_MOESM1_ESM.tif]

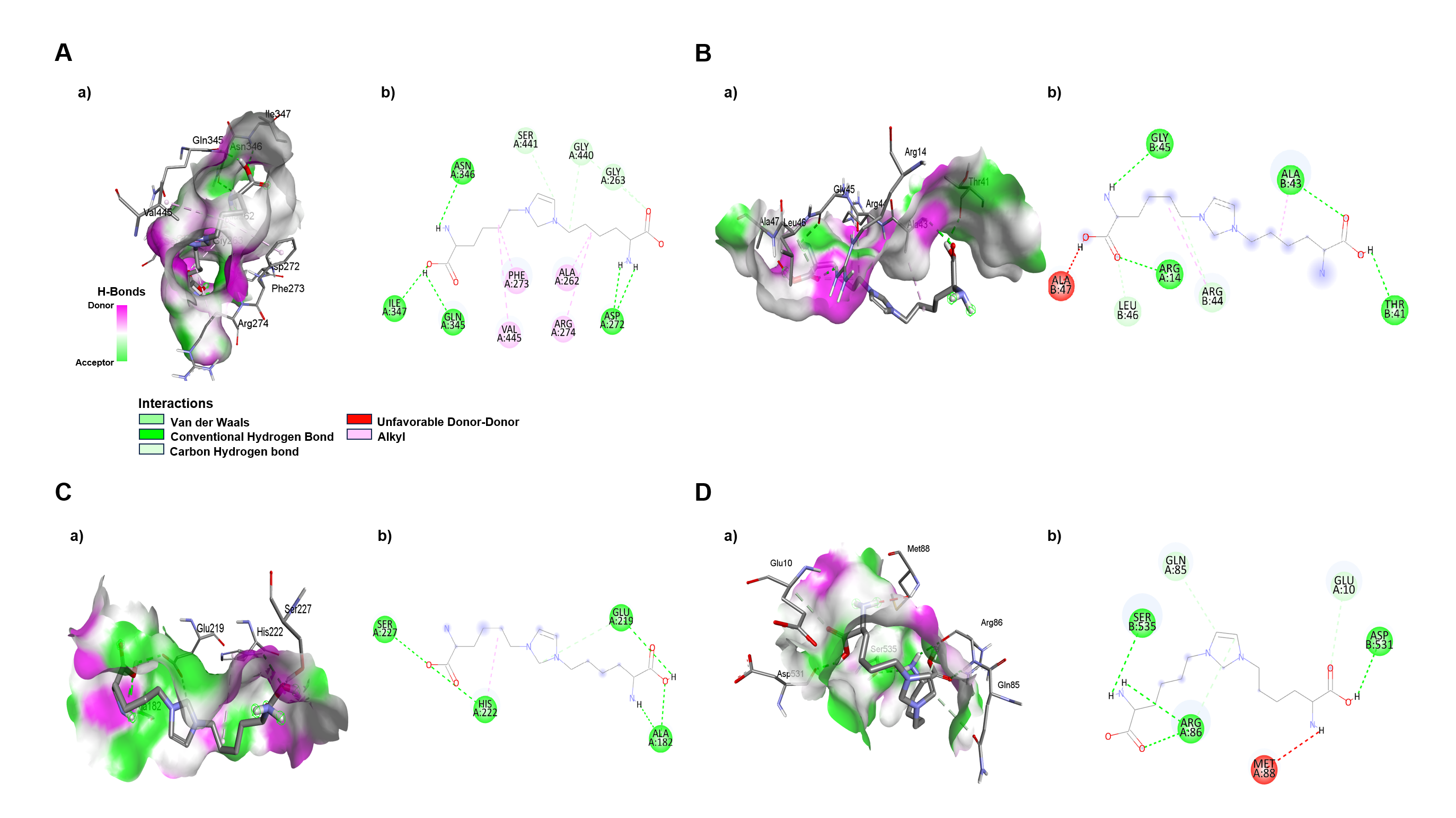

Supplement: Supplementary file 2 — Supplementary Figure 2. [file 41598_2024_52037_MOESM2_ESM.tif]

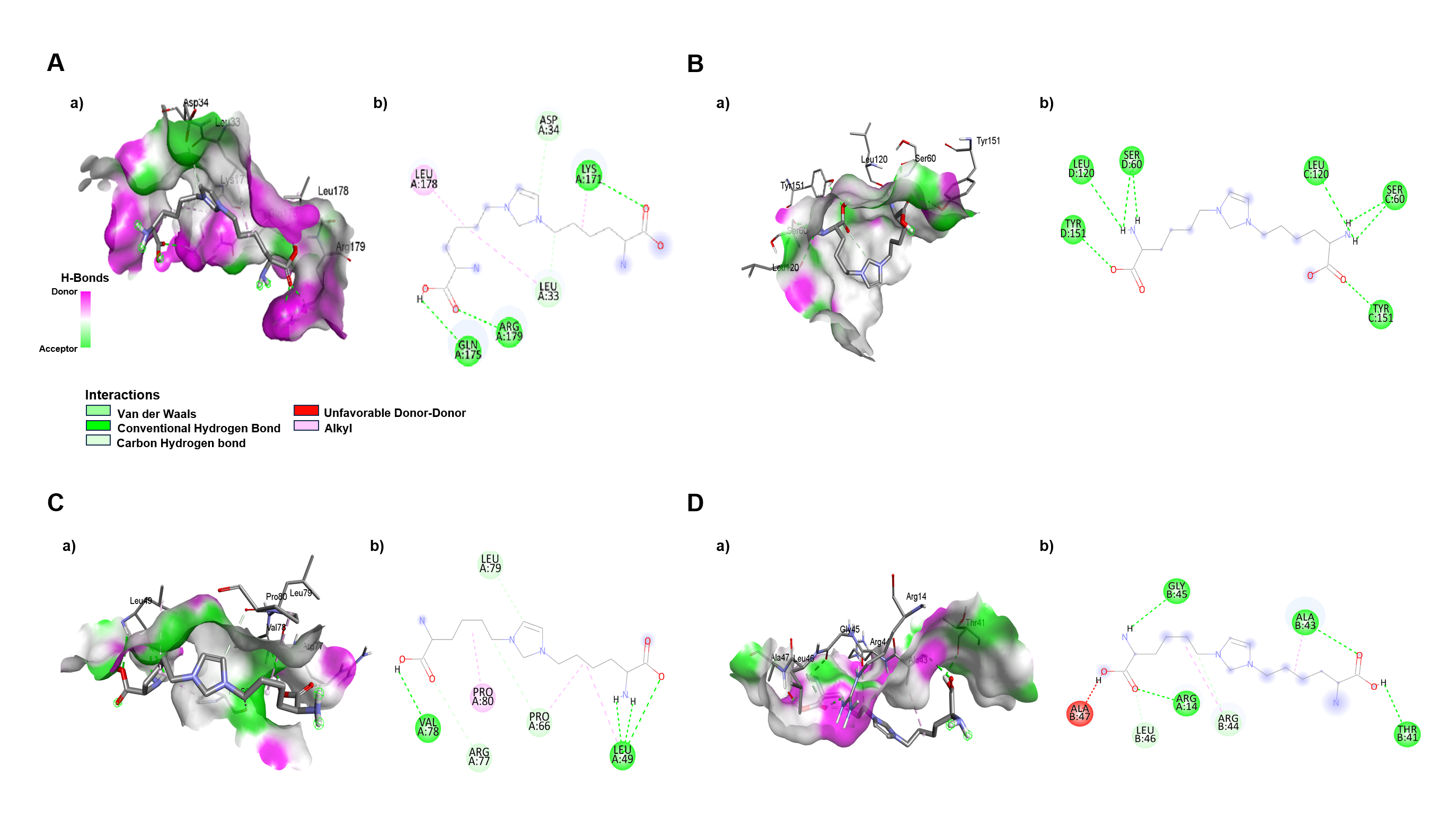

Supplement: Supplementary file 3 — Supplementary Figure 3. [file 41598_2024_52037_MOESM3_ESM.tif]

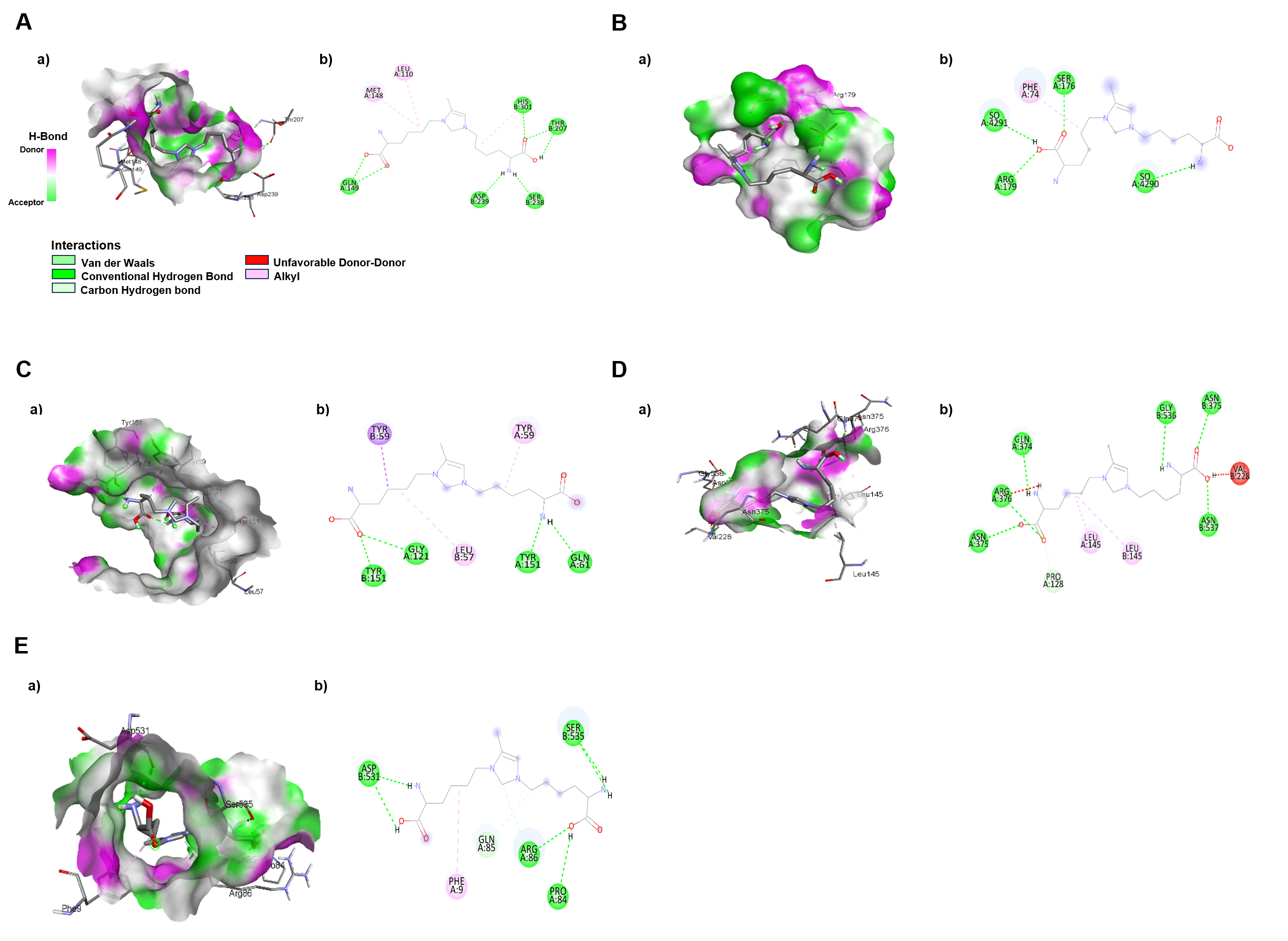

Supplement: Supplementary file 4 — Supplementary Figure 4. [file 41598_2024_52037_MOESM4_ESM.tif]

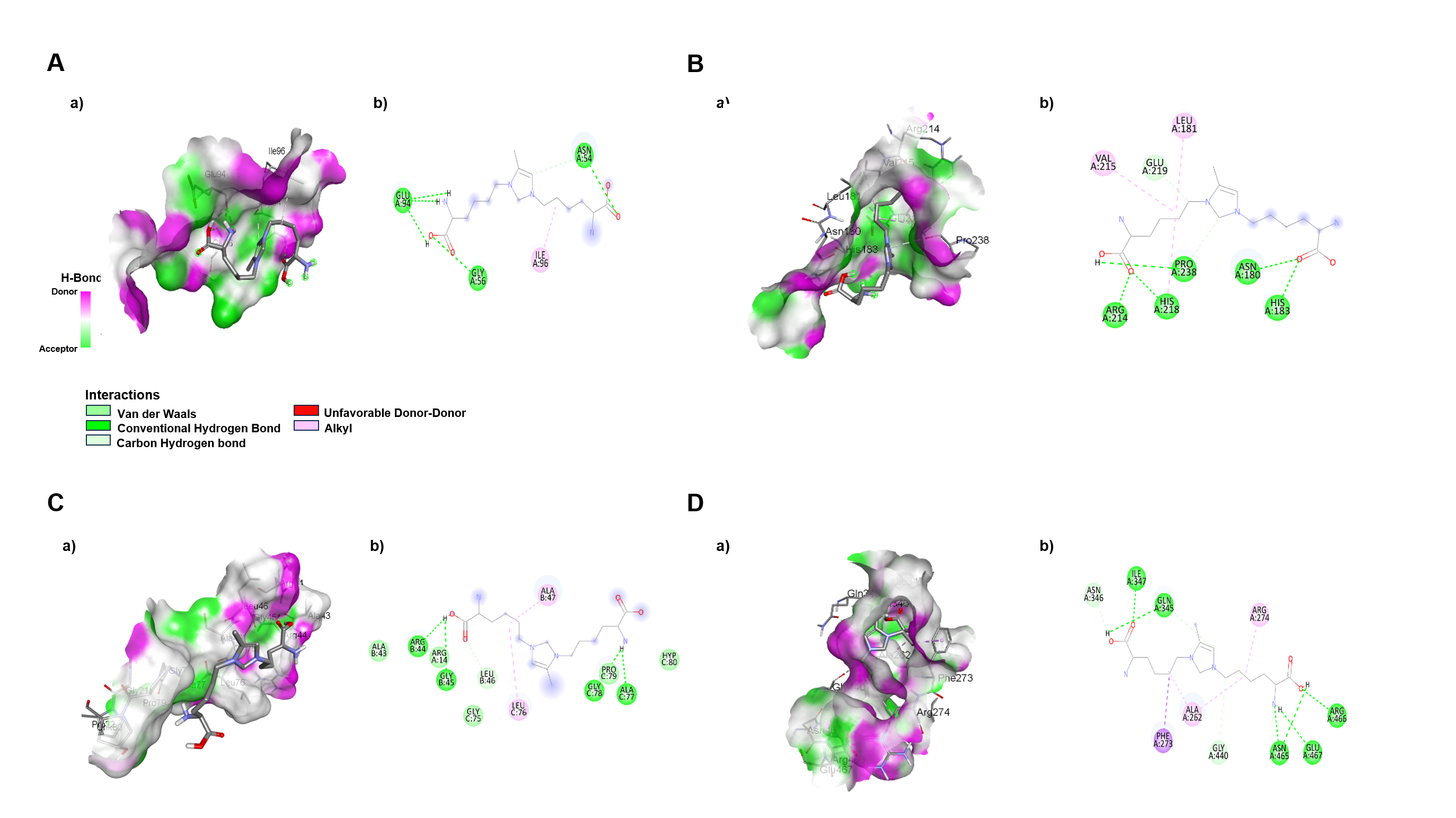

Supplement: Supplementary file 5 — Supplementary Figure 5. [file 41598_2024_52037_MOESM5_ESM.tif]

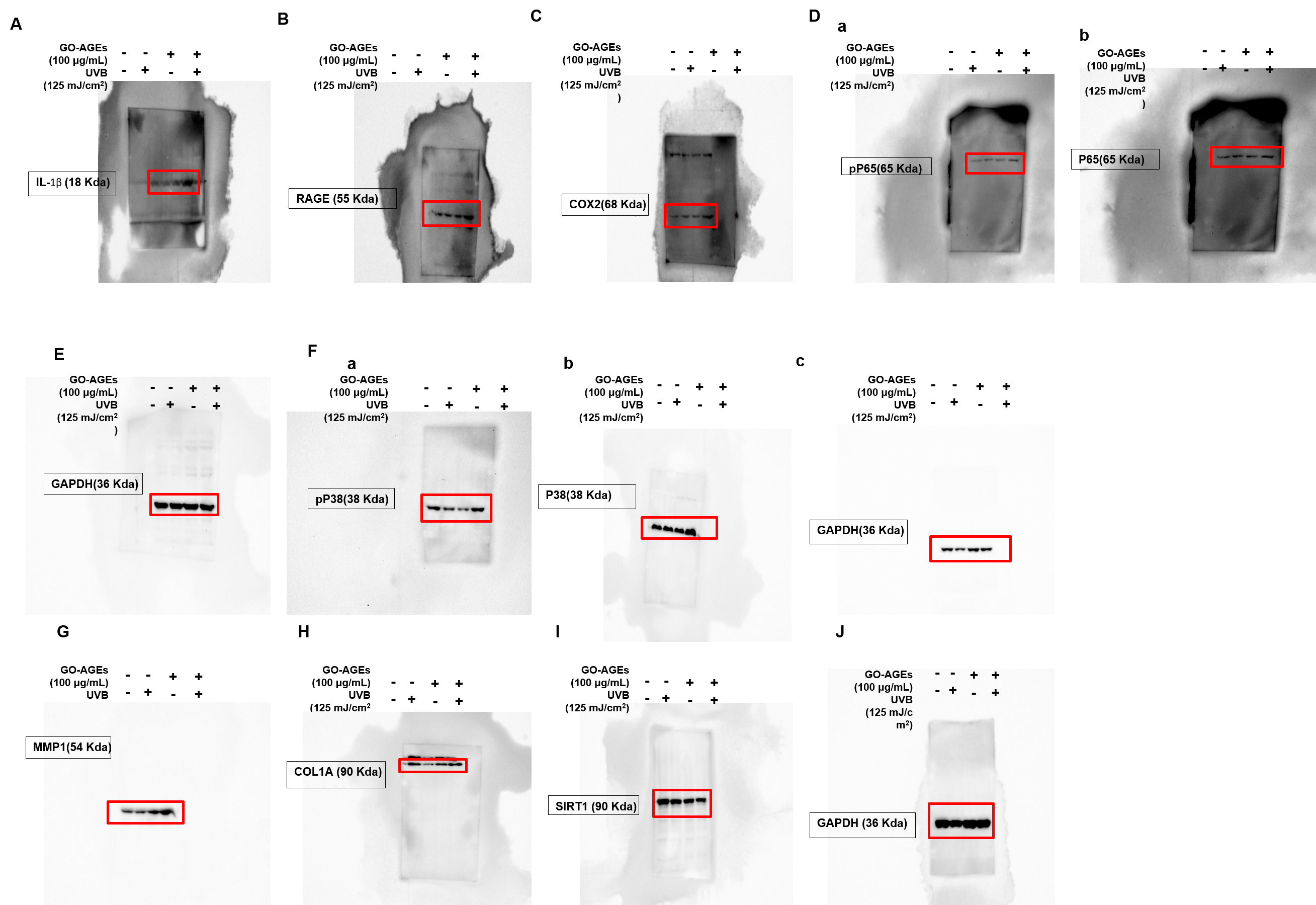

Supplement: Supplementary file 6 — Supplementary Figure 6. [file 41598_2024_52037_MOESM6_ESM.tif]
