# Supplementary material for: Glyoxal-derived advanced glycation end products (GO-AGEs) with UVB critically induce skin inflammaging: in vitro and in silico approaches
Source: Sci Rep. 2024 Jan 22;14:1843. doi: 10.1038/s41598-024-52037-z (PMC10800344; doi:10.1038/s41598-024-52037-z)
Supplement: Supplementary file 7 — Supplementary Legends. [file 41598_2024_52037_MOESM7_ESM.docx]

**SF.1.** Preliminary analysis of GO-AGEs in HaCaT cells for cytotoxicity and inflammatory cytokine secretion. A) MTT assay showing cytotoxic effects of different doses of GO-AGEs(50,100,250 μg/mL) in the presence or absence of UVB irradiation (125 mJ/cm^2^) along with the control group in HaCaT cells. B) ELISA results demonstrating the level of IL-1β in the conditioned medium of HaCaT cells induced by different doses of GO-AGEs (50,100 μg/mL) in the presence or absence of UVB irradiation(125 mJ/cm^2^). C) Western blot analysis showing the expression level of pP38, and P38 among others, after treatment with GO-AGEs(100 μg/mL) in the presence or absence of UVB irradiation(125 mJ/cm^2^) along with the control group in HaCaT cells. D) Quantification of the expression profiles of pP38, and P38,among others. E) Complex structure of a) GOLD with COL1A and b) the chemical structure of GOLD.F) Pocket-binding view (a) and different types of bonds found during the complex formation of (b) GOLD with COX2. Here, C is the control group, UVB group is the treatment group induced by UVB irradiation at 125 mJ/cm^2^; the GO-AGEs group represents the treatment group of GO-AGEs at 50,100,250 μg/mL; and the GO-AGEs + UVB treatment group represents GO-AGEs at 100 μg/mL in combination with UVB irradiation(125 mJ/cm^2^). Each value represents the mean ± SEM of triplicate experiments. (**) *p* < 0.01, and (***) *p* < 0.001 vs. the control group; and (#) *p* < 0.05, (##) *p* < 0.01, and (###) *p* < 0.001 vs. the UVB treatment group induced by UVB irradiation (125 mJ/cm^2^).

**SF.2.** The molecular docking study of GOLD with different proteins. The molecular docking study results showing pocket binding view(a) and different types of bonds (b) observed during complex formation of GOLD with A) SIRT1, B) IL-1β, C) MMP1, and D) P65

**SF.3.**The molecular docking study of GOLD with different proteins.The molecular docking study results showing pocket binding view (a) and different types of bonds observed during complex formation of (b) GOLD with A) IL-6, B)TNF-α, C) RAGE, and D) COL-1A

**SF.4.** The molecular docking study of MOLD with different proteins.The molecular docking study results showing pocket binding view (a) and different types of bonds observed during complex formation of (b) MOLD with A) IL-1β, B) IL-6, C) TNF-α, D) COX2, and E) P65

**SF.5.** The molecular docking study of MOLD with different proteins.The molecular docking study results showing pocket binding view (a) and different types of bonds observed during complex formation of (b) MOLD with A) RAGE B) MMP-1 C) COL-1A D) SIRT-1

**SF.6.** Full-length Western blot images representing the expression level of the following proteins in HaCaT cells (A-G) and NHDF cells (H-K). In HaCaT cell - A) IL-1β B) RAGE C) COX2 D) Phosphorylation of - (a) pP65 (b) P65 E) GAPDH F) Phosphorylation of -(a) pP38 (b) P38 (c) GAPDH and NHDF cells- G) MMP1 H) COL1A I) SIRT1 J) GAPDH
