# Supplementary material for: Glyoxal-derived advanced glycation end products (GO-AGEs) with UVB critically induce skin inflammaging: in vitro and in silico approaches
Source: Sci Rep. 2024 Jan 22;14:1843. doi: 10.1038/s41598-024-52037-z (PMC10800344; doi:10.1038/s41598-024-52037-z)
Supplement: Supplementary file 8 — Supplementary Information. [file 41598_2024_52037_MOESM8_ESM.pdf]

Representative full length images of western blot (obtained using Image Lab Softwear)(SF.6):

Western blotting was performed with three n=3, three independent replicates.some representative images were attached bellow:

HaCaT Cells:

GO-AGEs (100 µg/mL)<br>UVB (125 mJ/cm^2)	- - + +<br>- + - +	- - + +<br>- + - +	- - + +<br>- + - +
IL1B(18 KDa)	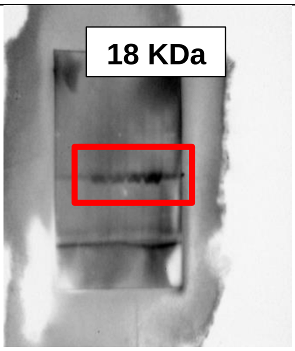	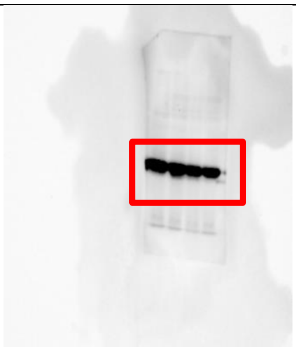	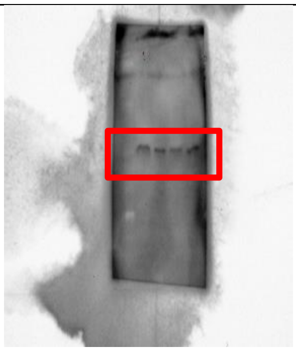
RAGE(55 KDa)	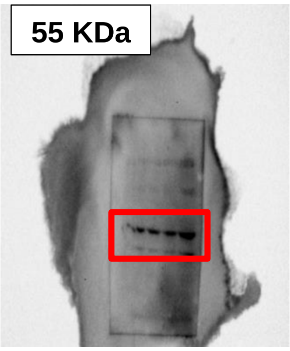	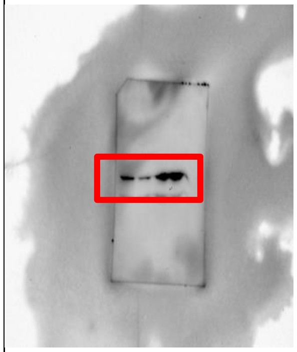	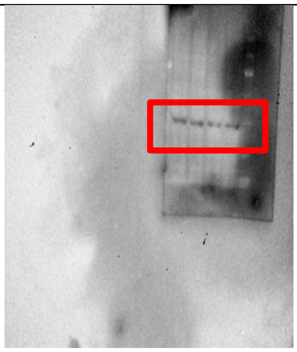
COX2(68 KDa)	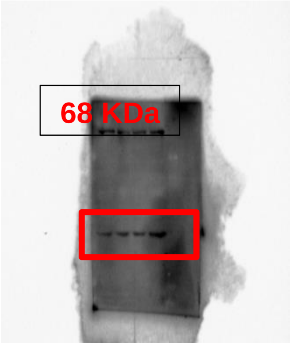	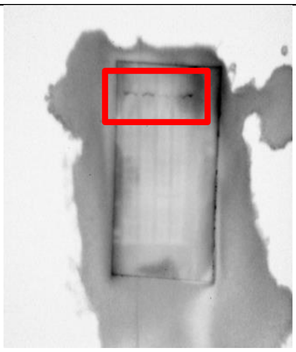	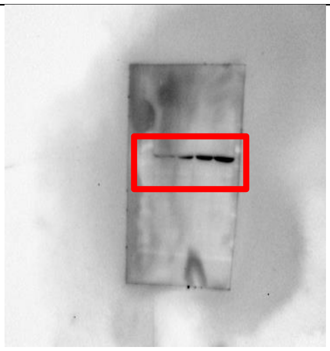

HaCaT Cells:

GO-AGEs (100 µg/mL)<br>UVB (125 mJ/cm^2)	- - + +<br>- + - +	- - + +<br>- + - +	- - + +<br>- + - +
pP65 (65 KDa)	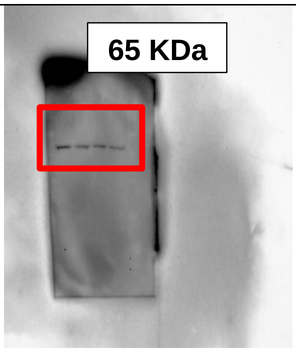	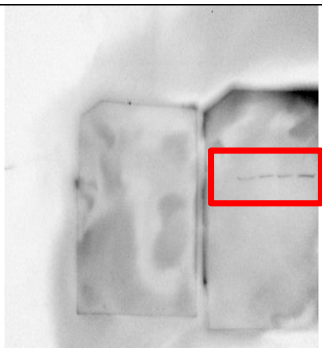	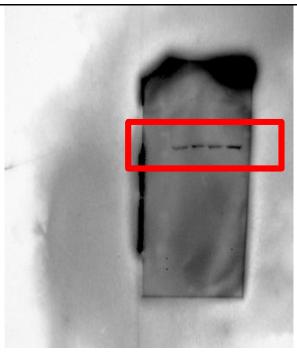
P65<br>(68 KDa)	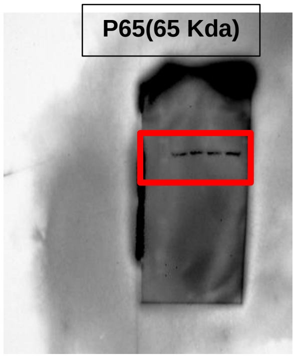	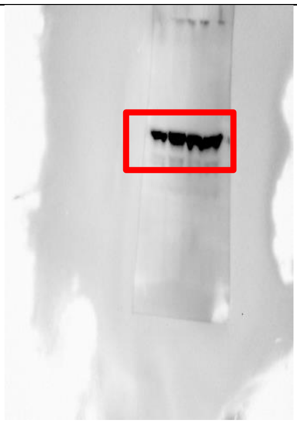	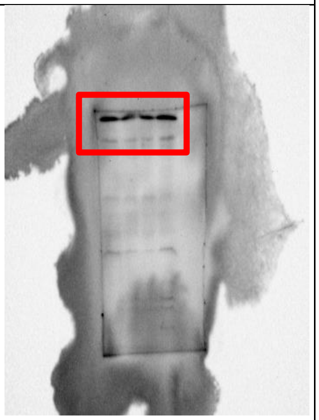
GAPDH<br>(36 KDa)	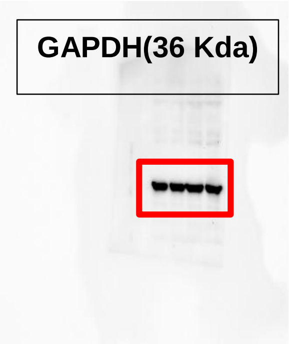	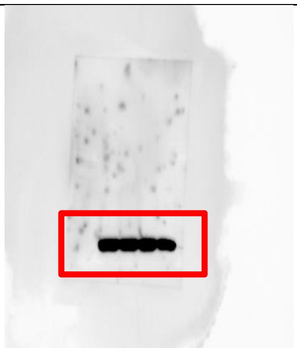	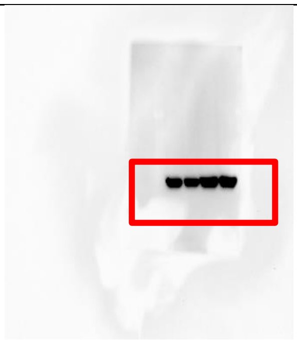

HaCaT Cells:

GO-AGEs (100 µg/mL)<br>UVB (125 mJ/cm^2)	- - + +<br>- + - +	- - + +	- - + +
**pP38 (38 KDa)**	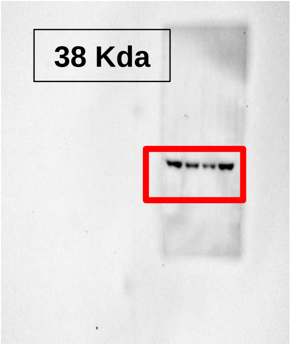	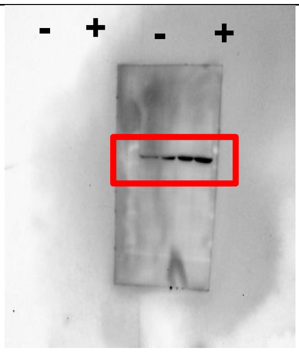	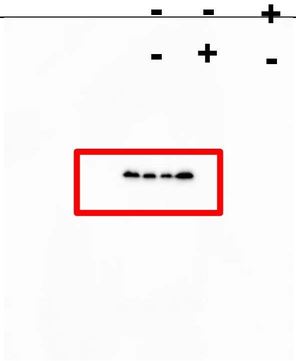
**P38(38 KDa)**	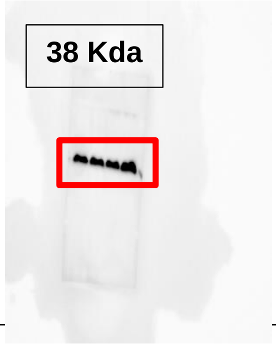	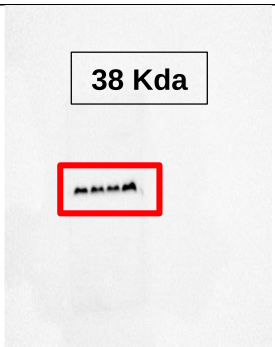	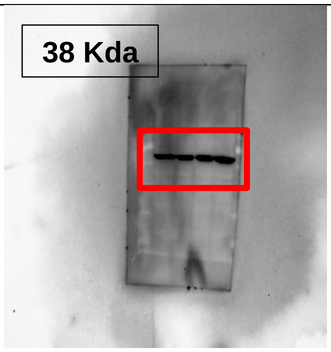
**GAPDH (36 KDa)**	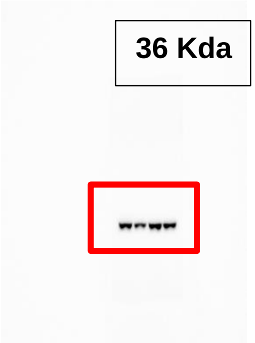	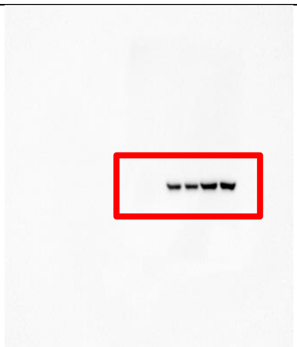	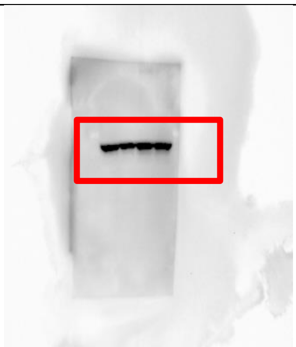

NHDF Cells:

GO-AGEs (100 µg/mL)<br>UVB (125 mJ/cm^2)	- - + +<br>- + - +	- - + +<br>- + - +	- - + +<br>- + - +
MMP1(54 KDa)	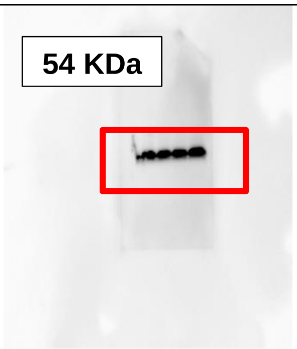	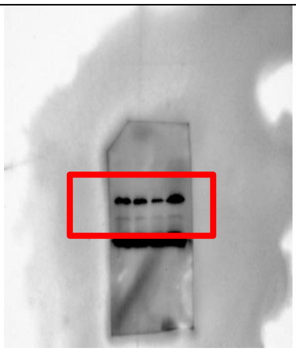	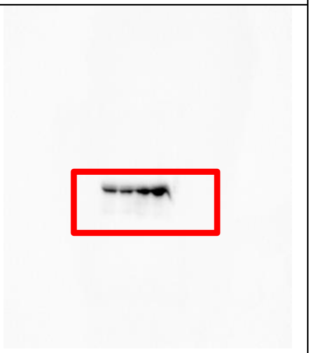
COL1A(90 KDa)	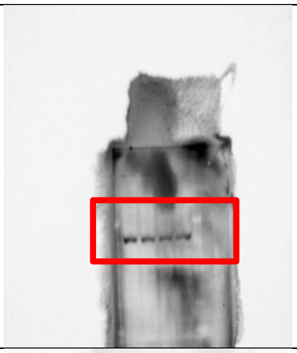	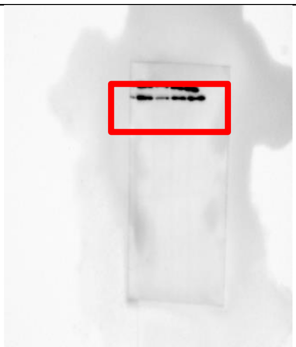	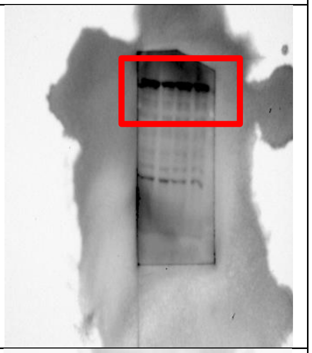
SIRT1(81 KDa)	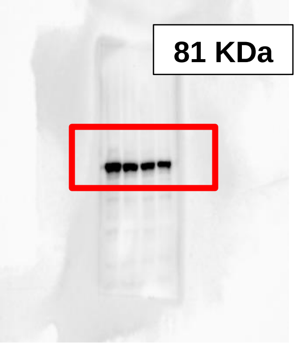	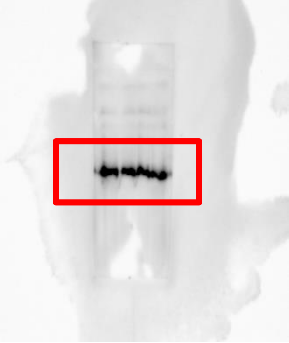	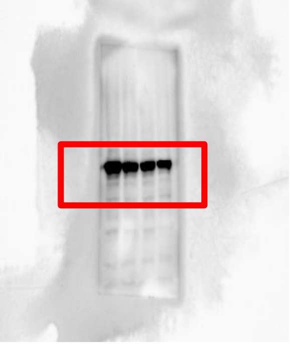

**GAPDH (36 KDa)**	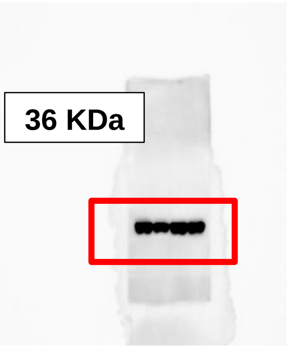	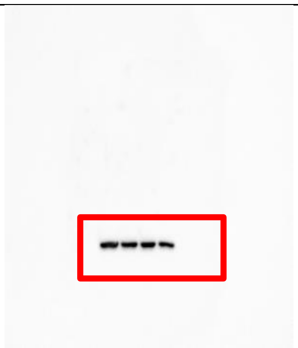	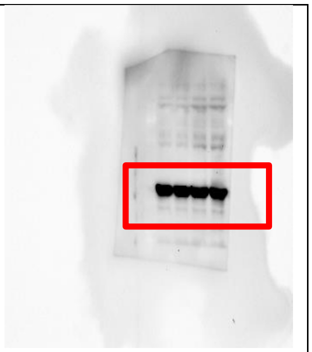
